# Regulation of cancer stem cells by CXCL1, a chemokine whose secretion is controlled by MCM2

**DOI:** 10.1186/s12885-024-12085-0

**Published:** 2024-03-07

**Authors:** Yeon-Jee Kahm, In-Gyu Kim, Rae-Kwon Kim

**Affiliations:** 1 Department of Radiation Biology, Environmental Safety Assessment Research Division, Korea Atomic Energy Research Institute, 111, Daedeok-Daero 989 Beon-Gil, Yuseong-Gu, 34057 Daejeon, Korea; 2 Department of Radiation Science and Technology, Korea University of Science and Technology, Yuseong-Gu, 34113 Daejeon, Korea

**Keywords:** MCM2, CXCL1, Cancer stem cells, EMT, Chemokine

## Abstract

**Background:**

A high expression pattern of minichromosome maintenance 2 (MCM2) has been observed in various cancers. MCM2 is a protein involved in the cell cycle and plays a role in cancer growth and differentiation by binding to six members of the MCM subfamily. The MCM protein family includes MCM2 through MCM7.

**Methods:**

MCM2 has shown high expression in both lung cancer stem cells (LCSCs) and glioma stem cells (GSCs). We investigated the characteristics of CSCs and the regulation of the epithelial-to-mesenchymal transition (EMT) phenomenon in LCSCs and GSCs by MCM2. Additionally, we explored secreted factors regulated by MCM2.

**Results:**

There was a significant difference in survival rates between lung cancer patients and brain cancer patients based on MCM2 expression. MCM2 was found to regulate both markers and regulatory proteins in LCSCs. Moreover, MCM2 is thought to be involved in cancer metastasis by regulating cell migration and invasion, not limited to lung cancer but also identified in glioma. Among chemokines, chemokine (C-X-C motif) ligand 1 (CXCL1) was found to be regulated by MCM2.

**Conclusions:**

MCM2 not only participates in the cell cycle but also affects cancer cell growth by regulating the external microenvironment to create a favorable environment for cells. MCM2 is highly expressed in malignant carcinomas, including CSCs, and contributes to the malignancy of various cancers. Therefore, MCM2 may represent a crucial target for cancer therapeutics.

**Supplementary Information:**

The online version contains supplementary material available at 10.1186/s12885-024-12085-0.

## Introduction

Minichromosome maintenance (MCM) proteins are known to be among the proteins regulating the cell cycle [1]. The MCM protein family consists of six types, from MCM2 to MCM7, and is a replication protein present in all eukaryotic cells from yeast to humans [2]. MCM2 plays an essential role in the initiation and elongation steps in DNA replication in the cell cycle [3]. Also, as a DNA replication licensing factor in the cell cycle, it is a protein that regulates only one replication in one cell cycle [4]. During cell proliferation, six types of MCM surround double-stranded DNA in the form of a heterohexamer complex at the origin throughout the M and G1 phases of the cell cycle [5, 6]. In this process, MCM proteins are located at the origin of chromatin replication along with the origin recognition complex (ORC), Cdc6, Cdt1, and ATP [7, 8]. During the S phase, MCM is phosphorylated by the kinases S-CDK and DDK, resulting in conformational modifications, and MCM2-7 associates with Cdc45 and GINS. The fully assembled Cdc45-MCM-GINS complex in the S phase is a functionally active, eukaryotic replicating helicase that unwinds the DNA double helix at the origin of replication. It has been confirmed that MCM2 is closely involved in the cell cycle, DNA damage repair, and drug resistance [9]. It has been reported that these characteristics can affect the maintenance of stem cell characteristics and self-renewal of tumors [10, 11]. However, studies on cancer stem cell (CSC) regulation by MCM2 have not been reported to date.

CSCs are known to have stem cell characteristics among cancer cells [12, 13]. CSCs are one of the biggest problems hindering cancer treatment, and many researchers have considered them as targets for treatment [14, 15]. CSCs are resistant to chemotherapy and radiation therapy [16, 17]. They also neutralize the efficacy of anticancer drugs by regulating the cell cycle [18, 19], and neutralize the efficacy of targeted anticancer drugs by utilizing various signaling mechanisms [20, 21]. They promote the growth of cancer by changing the microenvironment into a favorable environment for cancer cells to grow, as well as regulating the inside of the cell [22, 23]. At this time, CSCs control the microenvironment by secreting various cytokines including chemokines [24, 25]. The controlled microenvironment promotes cancer metastasis and protects cancer from immune cells by blocking or inactivating the access of surrounding immune cells [26, 27].

CXCL1 is one of the chemokines that act as chemoattractants for various immune cells and plays an important role in regulating infection and immune and inflammatory responses [28, 29]. However, in cancer, CXCL1 binds to its receptor CXCR2 and activates MAP kinases such as PI3k/Akt, ERK1/ERK2, and JNK [30, 31]. CXCL1 plays a role in angiogenesis and vasculogenesis and is known to be involved in tumor development in gastric, breast, colorectal, and lung cancers [32–35]. In animal studies, deficiency of CXCR2, a receptor for CXCL1, inhibits invasion of myeloid-derived suppressive cells and prevents colon cancer tumor formation, and CXCL1-mediated bone marrow cell invasion is known to be associated with treatment of breast cancer [36]. It has also been reported that autocrine and paracrine CXCL1 can promote tumor invasion and metastasis [37, 38].

In this study, we confirmed that CXCL1 is regulated by MCM2 in LCSC and GSC, and found that CSC characteristics and EMT phenomena are regulated through this signaling mechanism.

.

## Materials and methods

### Cell culture and sphere-formation assays

The A549 (CCL-185™) human lung cacer cell line was obtained from the ATCC and maintained in RPMI 1640 MEDIUM (Cat. No. SH30027.01; Hyclone, Cytiva, USA). The Human glioblastoma cell line, U-87 MG (30,014) was sourced from the Korea Cell Line Bank and cultured in DMEM/HIGH GLUCOSE (Cat. No. SH30243.01; Hyclone, Cytiva, USA). Both culture media were supplemented with 10% FBS (Cat. No. SH30919.03; Hyclone, Cytiva, USA) and 1% Penincillin-Streptomycin Solution (Cat. No. SV30010; Hyclone, Cytiva, USA). All cells were cultured in a humidified incubator at 37 ℃ with 5% CO_2_. During the sphere formation assay, cells were cultured in conditioned media (CM), made up of DMEM (DMEM-F12; Cat. No. 11320-033; Invitrogen; Thermo Fisher Scientific, Inc., Waltham, USA), basic fibroblast growth factor (20 ng/ml; Cat. No. 13256-029; Invitrogen; Thermo Fisher Scientific, Inc., Waltham, USA), epidermal growth factor (20 ng/ml; cat. no E9644; Sigma-Aldrich; Merck KGaA; Burlington, USA) and B27 Serum-Free Supplement (Cat. No. 17504-044; Invitrogen; Thermo Fisher Scientific, Inc., Waltham, USA).

### Neutralization assay

For the neutralization assay, the anti-CXCL1 antibody (Cat. No. MAB275; R&D SYSTEMS, Minneapolis, USA) was used at a concentration of 9 µg/mL. As a control, the normal mouse IgG1 antibody (Cat. No. sc-3877; Santa Cruz Biotechnology, Inc., Dallas, USA) was used. The experiment was conducted under the same cell culture conditions described earlier, and subsequent experiments or results were performed or obtained 24–48 h after the treatment with the antibodies.

### Antibodies

Antibodies against MCM2 (1:500) (Cat. No. 3619 S; Cell Signaling Technology, Inc., Danvers, USA), CD44 (1:1000) (Cat. No. 3570; Cell Signaling Technology, Inc., Danvers, USA), Sox2 (1:1000) (Cat. No. 3579; Cell Signaling Technology, Inc., Danvers, USA), Oct-4 (1:1000) (Cat. No. 2750; Cell Signaling Technology, Inc., Danvers, USA), Nanog (1:1000) (Cat. No. 4893; Cell Signaling Technology, Inc., Danvers, USA), Akt (1:1000) (Cat. No. 9272; Cell Signaling Technology, Inc., Danvers, USA), Phospho-Akt (S473) (1:1000) (Cat. No. 9271; Cell Signaling Technology, Inc., Danvers, USA), Phospho-Akt (T308) (1:1000) (Cat. No. 9275; Cell Signaling Technology, Inc., Danvers, USA), p38 (1:1000) (Cat. No. 9212; Cell Signaling Technology, Inc., Danvers, USA), Phospho-p38 (1:1000) (Cat. No. 9211; Cell Signaling Technology, Inc., Danvers, USA), ERK (1:1000) (Cat. No. 9102; Cell Signaling Technology, Inc., Danvers, USA), Phospho-ERK (1:1000) (Cat. No. 4370; Cell Signaling Technology, Inc., Danvers, USA), JNK (1:1000) (Cat. No. 9252; Cell Signaling Technology, Inc., Danvers, USA), Phospho-JNK (1:1000) (Cat. No. 9255; Cell Signaling Technology, Inc., Danvers, USA), ZEB1 (1:1000) (Cat. No. sc-25,388; Santa Cruz Biotechnology, Inc., Dallas, USA), SLUG (1:1000) (Cat. No. sc-166,476; Santa Cruz Biotechnology, Inc., Dallas, USA), SNAIL (1:1000) (Cat. No. sc-10,432; Santa Cruz Biotechnology, Inc., Dallas, USA), Twist (1:1000) (Cat. No. sc-15,393; Santa Cruz Biotechnology, Inc., Dallas, USA), β-catenin (1:1000) (Cat. No. sc-7963; Santa Cruz Biotechnology, Inc., Dallas, USA), ALDH1A1 (1:1000) (Cat. No. ab6192; Abcam, Cambridge, UK), ALDH1A3 (1:1000) (Cat. No. ab129815; Abcam, Cambridge, UK), E-Cadherin (1:1000) (Cat. No. ab15148; Abcam, Cambridge, UK), CD133 (1:1000) (Cat. No. ab19898; Abcam, Cambridge, UK), N-Cadherin (1:1000) (Cat. No. 610,920; BD Transduction, Franklin Lakes, New Jersey, USA), Vimentin (1:1000) (Cat. No. MA5-14564; Invitrogen; Thermo Fisher Scientific, Inc., Waltham, USA) were used for Western blot analysis and Immunocytochemistry assays.

### Small interfering RNA (siRNA) mediated knockdown of MCM2

A549 and U87 cells were transfected with siRNA targeting MCM2 (CACUCAGUACCUUGGAUCA, UGAUCCAAGGUACUGAGUG; Bioneer Corporation). Subsequently, 10 pmol siRNAs were transfected using Lipofectamine RNAi MAX reagent (Cat. No. 13-778-150; Invitrogen; Thermo Fisher Scientific, Inc., Waltham, USA). Stealth RNAi Negative Control Medium GC (Cat. No. 12935-300; Invitrogen; Thermo Fisher Scientific, Inc., Waltham, USA) was used as the negative control. Cells were incubated at 37 ℃ for at least 48 h after transfection.

### Western blotting

Cells were lysed using RIPA Lysis Buffer (Cat. No. 20–188; Millipore, Burlington, USA) containing phosphatase inhibitor cocktail tablets (Cat. No. 04906837001; Roche, Basel, Switzerland) and protease inhibitor cocktail tablets (Cat. No. 11,836,153,001; Roche, Basel, Switzerland). The protein concentration was determined using Protein Assay Dye Reagent Concentrate (Cat. No. #5,000,006; Bio-Rad Laboratories, Inc., Hercules, USA). For Western blot analysis, equal amounts of protein were separated on 8–15% sodium dodecyl sulfate (SDS)-polyacrylamide gels, and the separated proteins were transferred to Amersham™ Protran™ 0.2 μm NC (Cat. No. 10,600,001; Amersham™; Cytiva, USA) membranes. After blocking the transferred membranes with a solution of phosphate-buffered saline (PBS) containing non-fat milk (10%) and Tween 20 (0.1%) at room temperature for 1 h, the membranes were incubated with specific primary antibodies overnight in a cold chamber. Following washing with Tris-buffered saline (Cat. No. A0027; BIO BASIC, Markham, Canada), the membranes were treated with an HRP-linked secondary antibody (Anti-rabbit IgG; Cat. No. 7074 S, or Anti-mouse IgG; Cat. No. 7076 S; Cell Signaling Technology, Inc., Danvers, USA) for 2 h at room temperature. Protein bands were visualized using WESTERN BLOTTING LUMINOL REAGENT (Cat. No. sc-2048; Santa Cruz Biotechnology, Inc., Dallas, USA).

### Immunocytochemistry

Cells were grown onto glass coverlips in 35 mm plates and fixed with 4% paraformaldehyde (Cat. No. P2031; Biosesang, Seongnam, Republic of Korea) for 30 min at room temperature. After cell fixation, cells were incubated with antibodies in a solution of Tris-buffered saline (Cat. No. A0027; BIO BASIC, Markham, Canada) at 4 ℃ for overnight. The antibodies used were: MCM2 (1:400) (Cat. No. 3619 S; Cell Signaling Technology, Inc., Danvers, USA), ALDH1A1 (1:400) (Cat. No. ab6192; Abcam, Cambridge, UK), ALDH1A3 (1:400) (Cat. No. ab129815; Abcam, Cambridge, UK), CD133 (1:400) (Cat. No. ab19898; Abcam, Cambridge, UK), CD44 (1:400) (Cat. No. 3570; Cell Signaling Technology, Inc., Danvers, USA), E-Cadherin (1:400) (Cat. No. ab15148; Abcam, Cambridge, UK), N-Cadherin (1:400) (Cat. No. 610,920; BD Transduction, Franklin Lakes, USA), Vimentin (1:400) (Cat. No. MA5-14564; Invitrogen; Thermo Fisher Scientific, Inc., Waltham, USA). Staining was visualized using Alexa Fluor™ 488 donkey anti-mouse IgG (1:200) (Cat. No. A21202; Invitrogen; Thermo Fisher Scientific, Inc., Waltham, USA) and Alexa Fluor™ 488 donkey anti-rabbit IgG (1:200) (Cat. No. A21206; Invitrogen; Thermo Fisher Scientific, Inc., Waltham, USA). Nuclei were counterstained using 4,6-diamidino-2-phenylindole (DAPI solution; Cat. No. sc-24,941; Santa Cruz Biotechnology, Inc., Dallas, USA). Stained cells were analyzed using a Zeiss LSM510 Meta microscope (Carl Zeiss Micro Imaging GmbH, Göttingen, Germany).

### Single cell assay

Single cell experiments were set up with floating cells in an ultra-low adhesion 96-well plate (Cat. No. 3474; Corning, Inc., Corning, USA) with one or two cells distributed into each well. The cells were cultured in a humidified incubator with 5% CO_2_ at 37 ℃. The following day, wells with single cells in them were selected visually under a light microscope (magnification, x400), and after 10–14 days, spheres were quantified based on absolute count as well as diameter, and photographed using an inverted phase contrast microscope.

### Limited dilution assay

In a limited dilution assay, cells were plated in 200 µl spheroid formation assay medium in ultra-low adhesion 96-well plates. A total of 1, 10, 50, 100 or 200 cells/well were plated, with 48 wells for each starting density of cells. Oncospheres were analyzed using a light microscope (magnification, x400) after 10–14 days of incubation. A well with at least one spheroid with a diameter ≥ 100 μm was defined as a positive well, and the number of positive wells was counted.

### Invasion and migration assays

Migration assays were performed using an uncoated chamber (Cat. No. 3422; 8 μm pore; Corning, Inc., Corning, USA) and the ability of cells to migrate was measured. Invasion assays were performed by coating the chamber with Matrigel® according to the manufacturer’s protocol. The lower chamber of the Transwell inserts (Cell Biolabs) was filled with 800 µl RPMI 1640 supplemented with 10% FBS. In the upper chamber, 150 µl serum-free medium (Opti-MEM®; Cat. No. 31985-070; Invitrogen; Thermo Fisher Scientific, Inc., Waltham, USA) containing 2 × 10^5^ cells was added. The cells were incubated for 24 h at 37 ℃ in a humidified incubator with 5% CO_2_. Cells that had migrated/invaded to the bottom of the chamber were stained with crystal violet (Cat. No. HT90132-1 L; Sigma-Aldrich; Merck KGaA, Burlington, USA) and the cells were counted under a light microscope (x400, magnification).

### Wound healing assay

Cells were plated in a 60 mm culture dish and grown to 80% confluence. A wound was created by scraping the monolayer of cells with a 200 µl pipette tip in the middle. Floating cells were removed by washing with PBS and fresh medium containing 10% FBS was added. The doubling time of the A549 cells used was 24 h. Cells were incubated at 37 ℃ for 24 h, and imaged using phase-contrast microscopy (magnification, x400). The distance between the edges of the wounds shown in the image was measured randomly at three or more places and the mean of the three measurements were obtained.

### Colony-formation assay and irradiation

Cells were seeded at a density of 1 × 10^3^ cells per 35 mm cell culture dish (Cat. No. 430,165; Corning, Inc., Corning, USA), and then allowed to adhere for 24 h in a humidified incubator with 5% CO_2_ at 37 ℃. The following day, cells were irradiated with 3 Gy γ-radiation (KAERI). After 10–14 days, cells were stained for colonies (defined as clusters of ≥ 50 cells) with 0.5% crystal violet for 1 h at room temperature, and stained colonies were counted. Clonal survival rates are expressed as a percentage of the non-irradiated control group.

### Cytokine array

Human Cytokine Array Kit (Cat. No. ARY005B; R&D SYSTEMS, Minneapolis, USA) was used to evaluate the secreted factors regulated by MCM2 in lung cancer stem cells. A549 cell was transfected with siRNA targeting MCM2. All reagents should be brought to room temperature before use. Pipette 2 mL of blocking buffer into each well of the 4-Well Multi-dish and incubate for 1 h on a rocking platform shaker. Add 15 µl of reconstituted Human Cytokine Array Detection Antibody Cocktail to each prepared sample and incubate at room temperature for 1 h. After 1 h, vacate the 4-Well Multi-dish and add the prepared samples. Incubate overnight at 2–8 ℃ on a rocking platform shaker. Carefully remove each membrane and place into plastic containers with 20 mL of washing buffer. Wash each membrane with washing buffer for 10 min on a rocking platform shaker for 3 times. Pipette 2 mL of diluted Streptavidin-HRP into each well of the 4-Well Multi-dish. After 30 min incubation on a rocking platform shaker, wash each array with washing buffer for 3 times. Remove each membrane from the container and visualized by Chemi Reagent Mix.

### Kaplan-Meier plotter

Using the published genetic information system, Kaplan-Meier survival values were obtained (kmplot.com/analysis). This was based on results of mRNA gene chip analysis using tissues from lung cancer patients. The gene symbol used was MCM2. All conditions were set as total lung cancer patients.

### Statistical analysis

At least 3 independent experiments were repeated in all experiments, and the results of performances are expressed as the mean ± standard deviation. Each n value is written in the figure legend. Two-sided paired Student’s t-test was used to validate the data. *p* ≤ 0.05 was considered to indicate a statistically significant difference.

## Results

### MCM2 Expression in Lung Cancer Stem Cells and Its Prognostic Implications

**Fig. 1:**
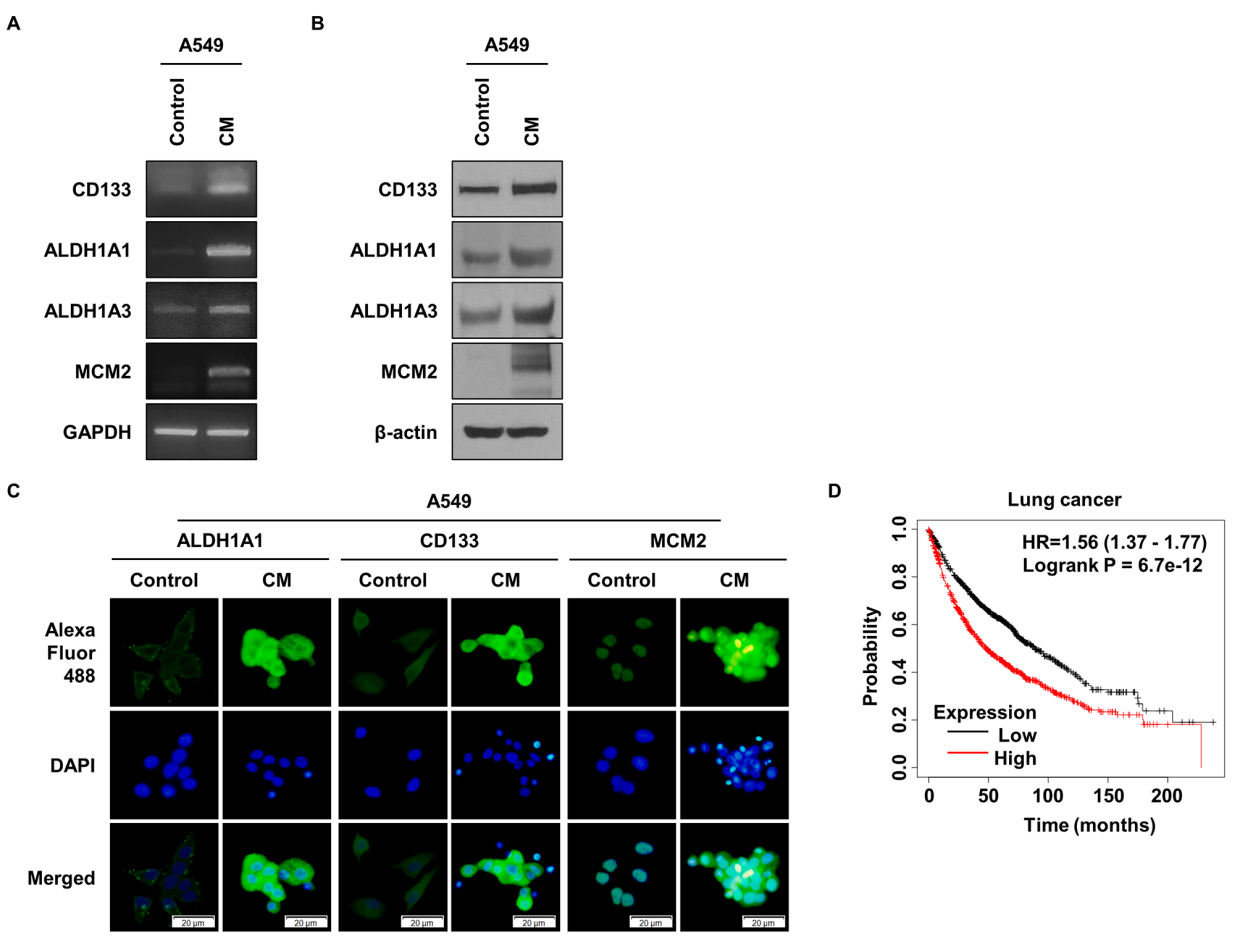
MCM2 Expression in Cancer Stem Cells and Prognostic Implications. (**A**) Comparison of RNA expression levels of CSC markers and MCM2 in A549 cells cultured in conditioned medium (CM). GAPDH was used as a loading control. (**B**) Comparison of protein expression of CSC markers and MCM2 in A549 cells cultured with CM. β-actin was used as a loading control. Full-length blots are presented in Supplementary Fig. 1A. (**C**) Comparison of expression levels of CSC marker protein and MCM2 using immunocytochemistry (ICC). (**D**) Kaplan-Meier survival curves for lung cancer patients according to MCM2 gene expression levels. Sample derive from the same experiment and that gels/blots were processed in parallel

ALDH1 is one of the important marker proteins for lung cancer stem cells (LCSCs) [39]. Through previous studies, we confirmed the level of expression of genes in ALDH1^+^ cells [40]. Furthermore, upon comparing our findings with those of other research groups, we consistently observed higher expression of MCM2. Another group’s research focused on gene expression in CD133^+^ cells, which are Glioblastoma Stem Cells (GSCs) [41]. Therefore, to explore the role of MCM2 in CSCs, we cultured A549 cells in conditioned media (CM) and utilized them for experimental purposes. Initially, we performed qPCR to analyze gene expression levels (Fig. 1A). The results revealed an upregulation of MCM2 expression along with the upregulation of the CSC markers, ALDH1, and CD133, in A549 cells grown under CM conditions. Figure 1B illustrates the Western blot (WB) results, which align with the trends observed in Fig. 1A. Additionally, to validate the WB results, we used immunocytochemistry (ICC) to observe the expression levels of ALDH1A1, CD133, and MCM2 in the A549 cell group cultured in CM (Fig. 1C). The proteins associated with CSC markers and MCM2 exhibited higher expression levels in A549 cells cultured under CM conditions. As a result of comparing the MCM2 expression pattern according to the culture dish environment, the expression of MCM2 was found to be higher on ultra-low attachment plates than on normal culture plates (Figure S2A). Figure 1D is a Kaplan Meier graph of lung cancer patients, confirming that the survival rate of patients with high MCM2 expression is low.

### MCM2 regulates lung cancer stem cells

**Fig. 2:**
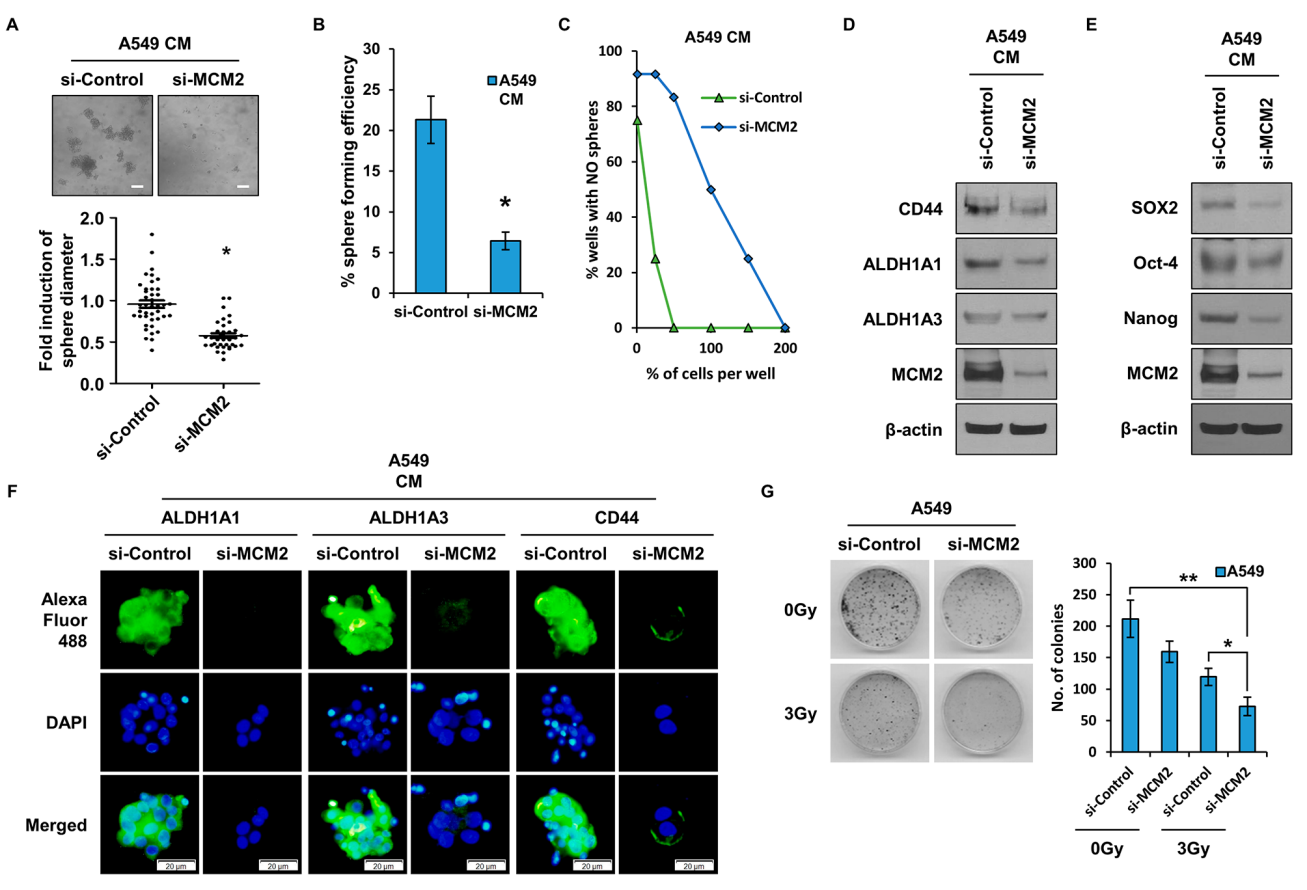
Impact of MCM2 Expression on Characteristics of LCSCs. (**A**) Comparative analysis of differences in sphere formation ability according to MCM2 expression. Comparison of sphere formation after 96 h of treatment of A549 cells with CM. (**B**) Single cell analysis of lung cancer stem cells (LCSCs) based on MCM2 expression. A549 cells were cultured in a 96-well plate to assess sphere-forming ability over a period of 10 days. (**C**) Limiting dilution assay to analyze LCSCs ability. Varying numbers of cells (1, 50, 100, 150, and 200) were plated in each well of a 96-well plate to compare sphere formation capacity. (**D**) Analysis of the expression amount of CSC marker proteins (CD44, ALDH1A1, ALDH1A3) according to MCM2 expression. Full-length blots are presented in Supplementary Fig. 1B. (**E**) Confirmation of the expression level of CSC regulatory proteins (SOX2, Oct-4, Nanog) according to MCM2 expression. Full-length blots are presented in Supplementary Fig. 1C. (**F**) Comparative analysis of the expression level of CSC marker proteins by ICC. (**G**) Colony formation assay to measure radioresistance based on MCM2 expression. To this end, 1 × 10 ^3^ cells were planted in each cell and irradiated with 3 Gy of radiation the next day. Sample derive from the same experiment and that blots were processed in parallel. Data represent the mean ± standard deviation of three replicates. Scale bar, 50 μm. *p˂0.005, **p˂0.05

Figure 2A presents the results to confirm the sphere formation ability, which is one of the characteristics of CSCs, by treating A549 cells with CM. When cancer cells are treated with CM, the population of CSC increases and spheres are formed. As a result of suppressing the expression of the MCM2 gene using si-RNA, it was confirmed that the sphere formation ability was significantly reduced compared to the control. As a result of confirmation in H460 cells, another cell line of lung cancer cells, results were similar to those of A549 (Figure S3A). A single cell assay was performed to confirm the self-renewal ability of CSCs (Fig. 2B). It was observed that sphere formation was significantly reduced in the group in which MCM2 expression was suppressed. As another experiment to confirm the self-renewal ability of CSCs regulated by MCM2, a limited dilution assay was carried out (Fig. 2C). It was confirmed from the results that when the MCM2 gene was inhibited, it was remarkably reduced, as found in the single cell assay. Figure 2D presents the expression of marker proteins of CSCs after suppressing the MCM2 gene. It was confirmed that the expression levels of CD44, ALDH1A1, and ALDH1A3, which are CSC marker proteins, were significantly decreased in the group in which the MCM2 gene was suppressed. In H460, the expression results of CD133, ALDH1A1, and ALDH1A3 were similar to those of A549 (Figure S3B). SOX2, Oct-4, and Nanog, known as CSC regulators, were also significantly reduced in the MCM2 gene suppression group (Fig. 2E). The expression of CSC marker proteins CD44, ALDH1A1, and ALDH1A3 by ICC was assessed and it was found that the expression of marker proteins was reduced in the group in which the MCM2 gene was suppressed, as shown in Fig. 2D (Fig. 2F). Another experiment was carried out to determine whether MCM2 is involved in CSCs having resistance to irradiation, and it was found that colony formation was inhibited by the MCM2 gene, as presented in Fig. 2G. A colony forming assay was then performed. Compared to the irradiated group, it was confirmed that colony formation was significantly reduced in the MCM2 gene suppression group.

### Regulation of EMT phenomenon and cell motility by MCM2 in lung cancer stem cells

**Fig. 3:**
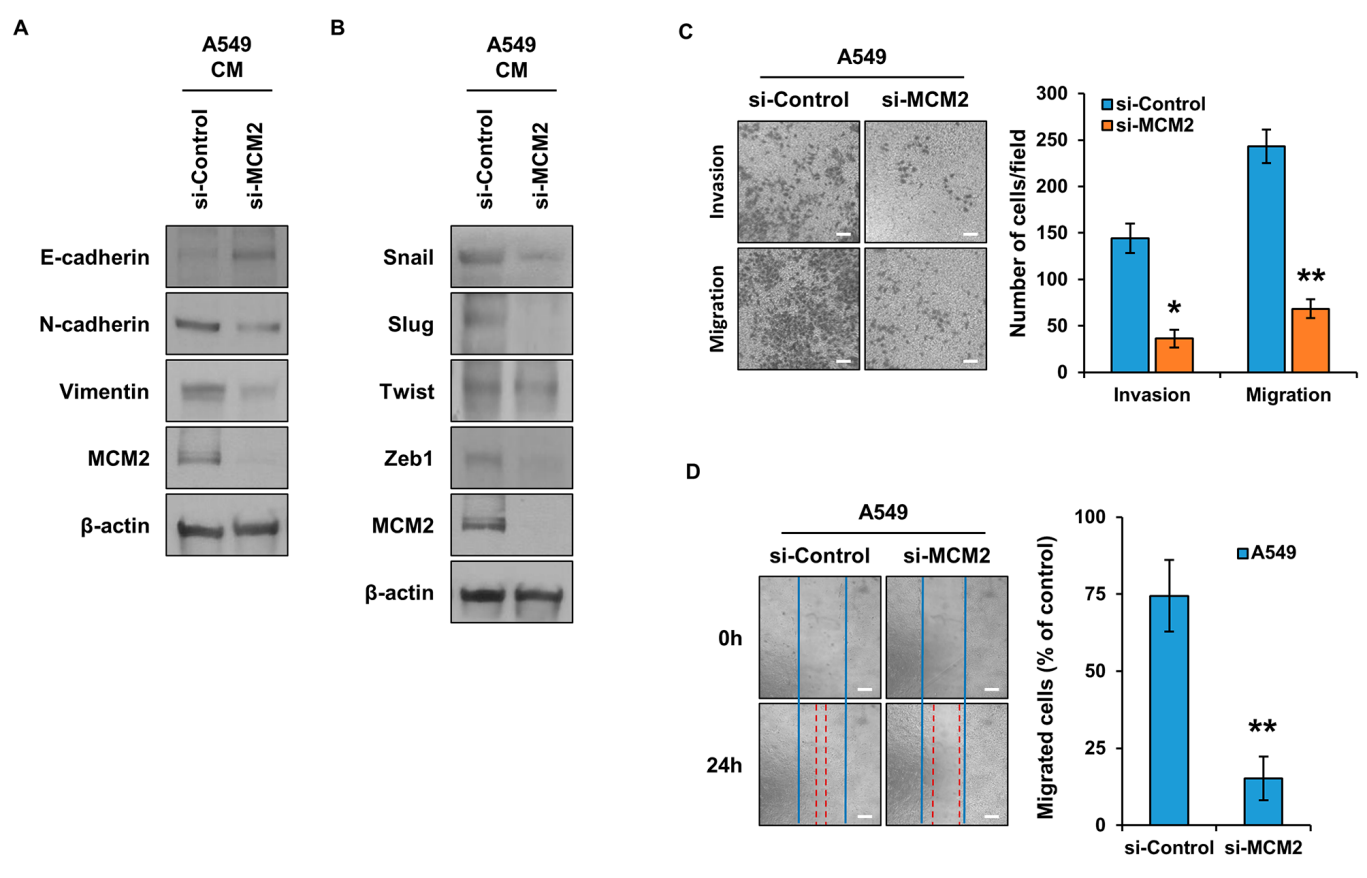
Influence of MCM2 Expression on EMT Marker Proteins and Cell Migration in LCSCs. (**A**) Confirmation of the expression levels of EMT marker proteins E-cadherin, N-cadherin, and vimentin according to the expression levels of MCM2. Full-length blots are presented in Supplementary Fig. 1D. (**B**) Expression levels of Snail, Slug, Twist, and Zeb1, which are EMT regulators. Full-length blots are presented in Supplementary Fig. 1E. (**C**) Confirmation of cell migration and invasion ability regulated by MCM2. (**D**) Measurement of cell migration ability according to the expression level of MCM2. Sample derive from the same experiment and that blots were processed in parallel. Data are presented as the mean ± standard deviation of three replicates. Scale bar, 50 μm. *p˂0.001, **p˂0.0005

CSCs are known to be closely related to the EMT phenomenon [12]. Therefore, we tried to confirm whether MCM2 is involved in the EMT phenomenon. The expression of MCM2 was inhibited, and the expression of E-cadherin, N-cadherin, and Vimentin, which are marker proteins of EMT, was respectively confirmed (Fig. 3A). As a result, in the MCM2 expression suppression group, E-cadherin, an epithelial marker protein, increased, and mesenchymal marker protein decreased. In H460 cells, inhibition of MCM2 expression increased the expression of E-cadherin and suppressed the expression of N-cadherin and Vimentin (Figure S3C). This indicates that MCM2 can regulate not only CSCs but also the EMT phenomenon. Figure 3B shows the results of evaluating the expression of Snail, Slug, Twist, and Zeb1, which are regulatory proteins of EMT. When the expression of MCM2 was inhibited, the expression of all the regulatory proteins of the EMT phenomenon was reduced. The most significant feature of the EMT phenomenon is that cell motility increases. We analyzed the migration ability to accurately assess whether MCM2 regulates the EMT phenomenon. A migration and invasion assay using a Boyden chamber was used to evaluate the migration ability of cells according to the expression of MCM2 gene (Fig. 3C). As a result, in the group in which the MCM2 gene was suppressed, both the cell migration ability and the invasiveness were reduced. Inhibition of MCM2 expression also reduced cell migration and invasion ability in H460 cells (Figure S3D). In addition, the migration ability of the cells was analyzed through a wound healing assay, and the results showed that the migration ability was reduced in the MCM2 gene suppressed group, as shown in Fig. 3C (Fig. 3D).

### Regulation of secreted cytokines by MCM2 in lung cancer stem cells and their impact on cell behavior

**Fig. 4:**
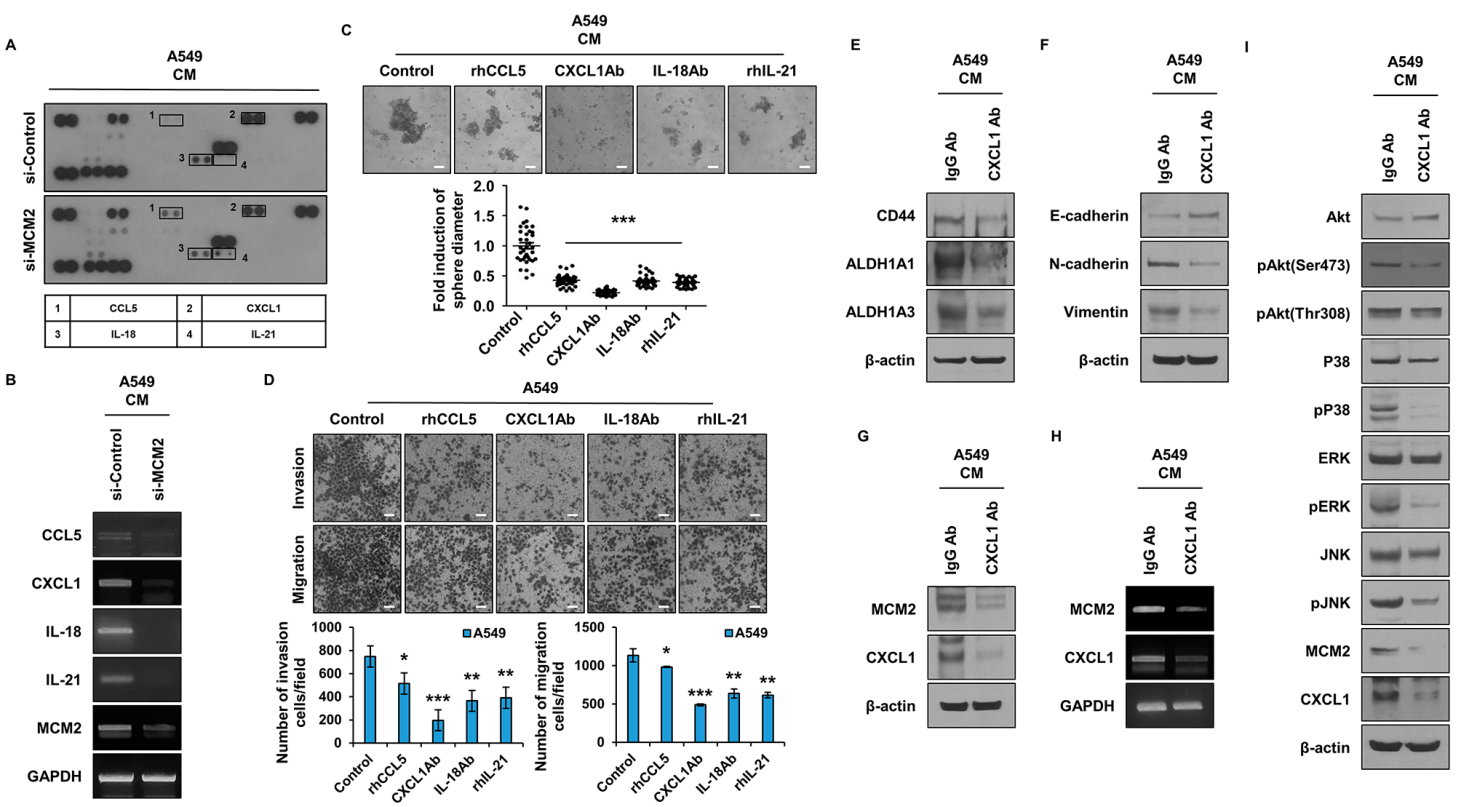
Regulation of Secreted Factors by MCM2 in LCSC. (**A**) Identification of secreted factors regulated by MCM2 using cytokine array. (**B**) Gene expression of secreted factors (CCL5, CXCL1, IL-18, IL-21) according to the expression level of MCM2. (**C**) Comparative analysis after conducting sphere-forming assay by treating with neutralizing antibodies of each secreted factor. (**D**) Comparative analysis of cell migration and invasion ability by treatment with neutralizing antibodies of each secreted factor. (**E**) Expression levels of CSC marker proteins after treatment with CXCL1 neutralizing antibody. Full-length blots are presented in Supplementary Fig. 1F. (**F**) Expression levels of EMT marker proteins after treatment with CXCL1 neutralizing antibody. Full-length blots are presented in Supplementary Fig. 1G. (**G**) Confirmation of the protein expression levels of MCM2 and CXCL1 after treatment with CXCL1 neutralizing antibody. Full-length blots are presented in Supplementary Fig. 1H. (**H**) Confirmation of gene expression levels of MCM2 and CXCL1 after treatment with CXCL1 neutralizing antibody. (**I**) Investigation of the CXCL1 signaling mechanism after inhibiting the action of CXCL1. Full-length blots are presented in Supplementary Fig. 1I. Sample derive from the same experiment and that gels/blots were processed in parallel. Data are presented as the mean ± standard deviation of three replicates. Scale bar, 50 μm. *p˂0.05, **p˂0.005, ***p˂0.0005

CSCs are known to secrete various cytokines [12, 13]. Therefore, we aimed to identify the secreted factors regulated by MCM2 (Fig. 4A). When MCM2 gene was suppressed, we observed a decrease in the secretion of CCL5, CXCL1, IL-18, and IL-21 in the conditioned medium (CM) (Fig. 4A). The reduced cytokines in si-MCM2-treated cells were further evaluated using qPCR (Fig. 4B), confirming that all cytokine genes were decreased in the group with inhibited si-MCM2 gene expression. To investigate the effects of these secreted factors on cell behavior, we performed sphere-forming assays after treating cells with neutralizing antibodies against each cytokine (Fig. 4C). The results showed that the CXCL1 neutralizing antibody significantly inhibited sphere formation, and the other neutralizing antibodies also suppressed sphere-forming ability. To assess the impact of these factors on cell motility and invasion, we performed experiments using Boyden chambers (Fig. 4D). As a result of measuring the migration and invasion ability of cells treated with each neutralizing antibody, the overall trend was decreased as shown in Fig. 4C. In particular, cell movement was significantly reduced in the group treated with CXCL1 neutralizing antibody. As a result of confirming the expression of CXCL1 according to MCM2 expression by ICC, intracellular CXCL1 expression was also regulated by MCM2 (Figure S4A). Furthermore, we examined the expression of CSC marker proteins, ALDH1A1, ALDH1A3, and CD44, after inhibiting CXCL1 using neutralizing antibodies (Fig. 4E). In the group in which CXCL1 was inhibited, the expression of CSC marker proteins was reduced. In Fig. 4F, we evaluated the expression of EMT marker proteins, E-cadherin, N-cadherin, and Vimentin, after inhibiting CXCL1 with neutralizing antibodies. As shown in Fig. 4F, the expression of epithelial marker E-cadherin increased, and the expression of mesenchymal markers N-cadherin and Vimentin decreased. To investigate the correlation between CXCL1 and MCM2, we observed the expression of MCM2 after treating cells with neutralizing antibodies against CXCL1 (Fig. 4G). As a result, the expression of MCM2 and CXCL1 were decreased in the neutralizing antibody-treated group. To confirm the change at the gene level, qPCR was performed (Fig. 4H). As a result, the expression of MCM2 was suppressed in the group treated with the neutralizing antibody of CXCL1, and the expression of CXCL1 was also decreased. CXCL1 has been reported to be involved in PI3K-AKT and MAPK signaling in cells [30, 31]. Thus, an experiment was carried out to determine whether CXCL1 uses the MAPK signaling mechanism (Fig. 4I). When A549 cells were treated with CXCL1 neutralizing antibody, it was confirmed that the activities of AKT, P38, ERK and JNK, which are MAPKs, were reduced.

### MCM2’s involvement in GSCs regulation and EMT regulation in glioma

**Fig. 5:**
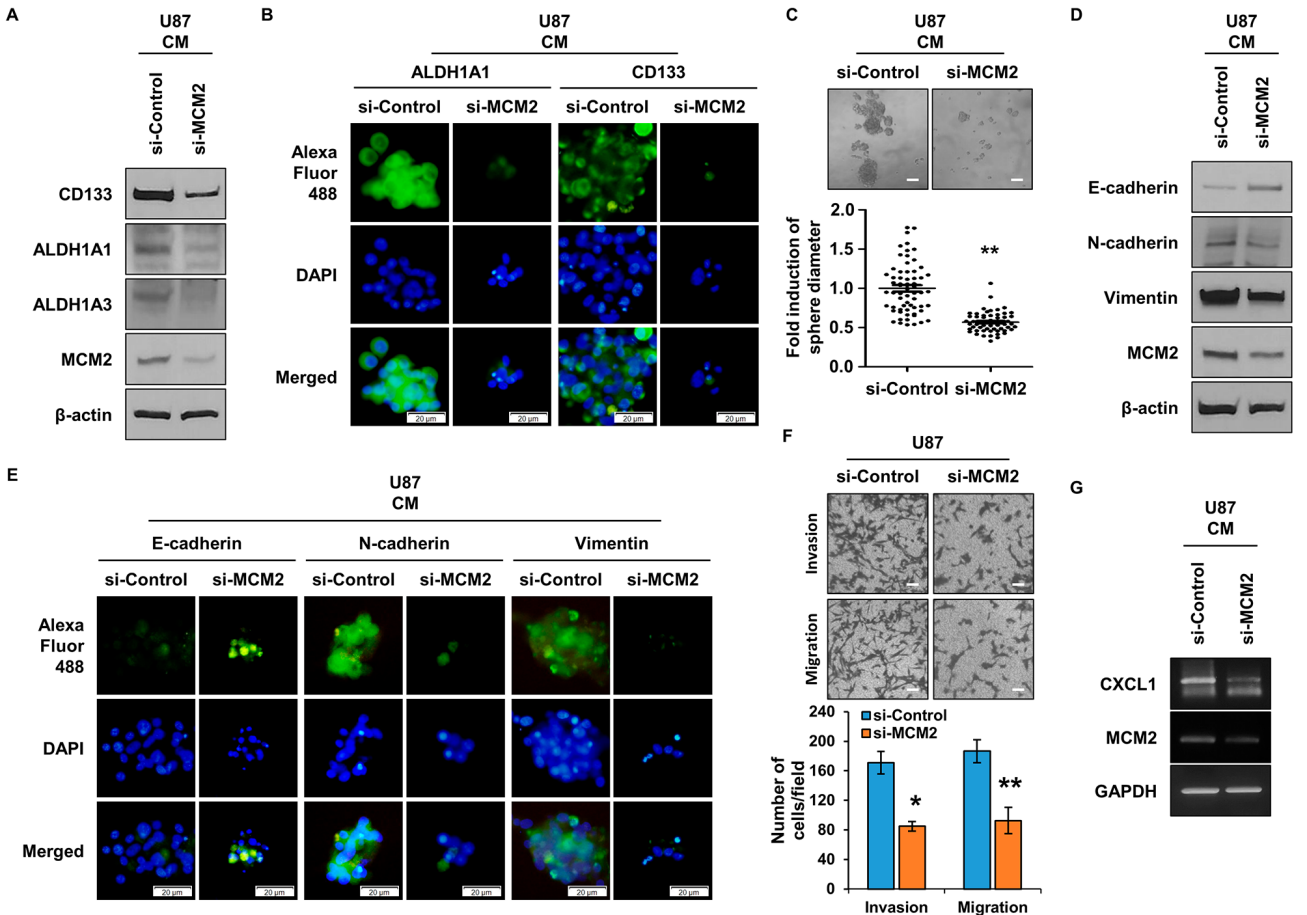
Regulation of GSC and EMT Properties by MCM2 Inhibition. (**A**) Expression of GSC marker proteins by MCM2 gene suppression in GSCs. Full-length blots are presented in Supplementary Fig. 1J. (**B**) Confirmation of marker protein expression in GSCs using ICC. (**C**) Comparison of differences in sphere formation according to the expression level of MCM2. (**D**) Comparison of expression of EMT marker proteins by MCM2 gene suppression. Full-length blots are presented in Supplementary Fig. 1K. (**E**) Confirmation of EMT marker proteins expression using ICC. (**F**) Analysis of cell migration and invasion ability after inhibiting the expression of MCM2. (**G**) Analysis of the regulation of CXCL1 expression by MCM2 in GSCs. Sample derive from the same experiment and that gels/blots were processed in parallel. Data are presented as the mean ± standard deviation of three replicates. Scale bar, 50 μm. *p˂0.001, **p˂0.005

MCM2 is a gene commonly overexpressed in cells overexpressing the LCSC marker protein and cells overexpressing the GSC marker protein [40, 41]. Therefore, we sought to analyze how MCM2 affects GSCs. As shown in Fig. 5A, glioma cell line U87 cells were treated with si-RNA to inhibit MCM2 expression, and then the cells were harvested to confirm the expression of the CSC marker protein. As a result, it was confirmed that the expression of marker proteins ALDH1A1, ALDH1A3, CD133, and CD44 was decreased. The same results were obtained in U373, another glioma cell line (Figure S5A). Modulation of marker proteins was further confirmed by immunofluorescence staining (Fig. 5B). Figure 5 C shows the results of measuring the sphere formation ability, which is a characteristic of CSCs. It is seen that the sphere formation ability decreased when the expression of MCM2 was decreased in GSCs. When MCM2 expression was suppressed in U373 cells, the sphere forming ability was significantly reduced (Figure S5B). As a result of determining the expression of E-cadherin, N-cadherin, and Vimentin, which are EMT marker proteins, it was confirmed that the EMT phenomenon was regulated in the group in which MCM2 expression was suppressed (Fig. 5D). As the expression of MCM2 was suppressed, E-cadherin increased and N-cadherin and Vimentin decreased in U373 cells (Figure S5C). From the evaluation of the EMT marker proteins using immunofluorescence staining, the results shown in Fig. 5D were confirmed (Fig. 5E). The migration ability of cells exhibiting the EMT phenomenon was then measured using a Boyden chamber (Fig. 5F). It was confirmed that the ability of cells to migrate and penetrate was significantly reduced in the group in which the expression of MCM2 was suppressed. In U373 cells, cell migration and invasion abilities were also significantly reduced by inhibiting the expression of MCM2 (Figure S5D). In the case of LCSC, it was confirmed that CXCL1 was secreted to regulate CSC and EMT phenomena through autocrine action. Therefore, as a result of evaluating the expression of CXCL1 in GSCs, it was confirmed that the expression of CXCL1 in GSCs is regulated by MCM2 (Fig. 5G).

## Discussion

In this paper, it was confirmed that MCM2 was overexpressed in LCSC and GSC, and it was also demonstrated that MCM2 regulates CSC. LCSC and GSC marker proteins ALDH1, CD44, and CD133 were regulated by MCM2, as were CSC regulators Sox2, Oct4, and Nanog. It has been reported that MCM2 regulates stem cells by combining with H3-H4 [42]. It has been reported that it binds to histone and regulates stem cell differentiation by regulating Sox2, Oct4, and Nanog, which are regulators of stem cells. Additionally, it was shown that MCM2 was overexpressed in cancer [43], and it was reported that MCM2 plays an important role in breast CSCs and liver CSCs [44, 45]. We plan to further assess whether MCM2’s regulation of CSCs is regulated through histone binding. It was confirmed that MCM2 not only regulates CSCs, but also changes E-cadherin, N-cadherin, and Vimentin, which are marker proteins related to EMT, and regulates Snail, Slug, Zeb1, and Twist, which are regulatory proteins. This phenomenon suggests that cancer stem cells had the characteristics of mesenchymal cells, but were changed to the characteristics of epithelial cells by inhibition of MCM2. EMT phenomenon occurs in cells treated with CM, but when MCM2 expression is suppressed, MET phenomenon occurs. MCM2 is a protein involved in the cell cycle and is known to be involved in cell growth and division, but is also emerging as an important marker in cancer. Through this study, it was confirmed that the radioresistance of lung cancer cells was suppressed by the regulation of MCM2 expression. MCM2 is not just a marker, but may be a therapeutic target.

In addition, this study confirmed that one of the chemokines, CXCL1, was regulated by MCM2. Through this, it was found that MCM2 acts not only intracellularly but also extracellularly by regulating the secretion of CXCL1. In general, CXCL1 is known to be involved in the activation of MAP kinases such as PI3K/Akt, ERK, and JNK through the receptor CXCR2. It was confirmed that the activity of MAP kinase was affected through CXCL1 autocrine regulation by MCM2. However, the signaling mechanisms involved in the regulation of CXCL1 by MCM2 require further investigation. Future studies will report on the relationship between MCM2 and CXCL1.

In conclusion, we revealed that MCM2 is involved in the regulation of LCSCs and GSCs by regulating the expression of CXCL1. However, we cannot conclude whether MCM2 is regulated through CXCL1 or through direct binding to histone. This will be revealed through further experiments. MCM2 is overexpressed in many cancers and is known to be an important factor in some CSCs. We confirmed that MCM2 is not only an important factor in LSCS and GSC, but also modulates the ability to resist radiation. Therefore, MCM2 is thought to be an important novel target in cancer therapy.

### Limitations

This study has several limitations. First, the carcinomas identified are limited to lung cancer and GBM. The effect of MCM2 on other cancer types needs to be confirmed. Second, experiments using cell lines may not be representative of all lung cancers and GBMs. However, through additional cell line experiments, it is believed that this problem has been alleviated to some extent. Third, malignant transformation of cancer cells was confirmed due to the identified secreted factors, but the effect on the surrounding environment was not confirmed. The effects on immune cells or normal cells existing around cancer will need to be studied in the future.

### Electronic supplementary material

Below is the link to the electronic supplementary material.

Supplementary Material 1

## Data Availability

Kaplan-Meier results were confirmed at https://kmplot.com/analysis. CD133^+^ Glioma microarray results were obtained from Yan et al. (Ref No. 41) in the paper.
