# Supplementary material for: Regulation of cancer stem cells by CXCL1, a chemokine whose secretion is controlled by MCM2
Source: BMC Cancer. 2024 Mar 7;24:319. doi: 10.1186/s12885-024-12085-0 (PMC10921750; doi:10.1186/s12885-024-12085-0)
Supplement: Supplementary file 1 — Supplementary Material 1 [file 12885_2024_12085_MOESM1_ESM.pdf]

Figure S1.

A

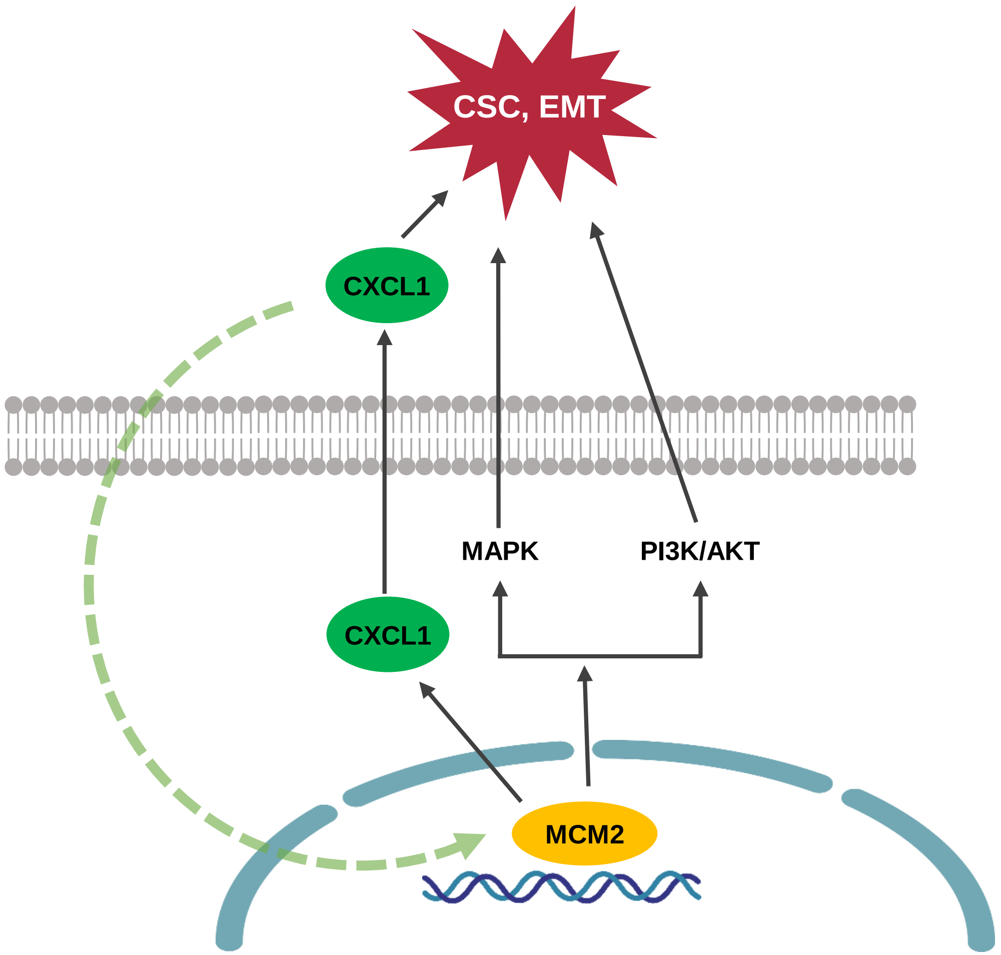

Supplymentary Figure 1. Schematic model of CSC associated. (A) It was confirmed that CSC and EMT are occurring due to PI3K/AKT signals and MAPK signals regulated by MCM2. In addition, it affects CSC and EMT by regulating the secretion factor CXCL1, and regulates MCM2 again by autocrine loop.

Figure S2.

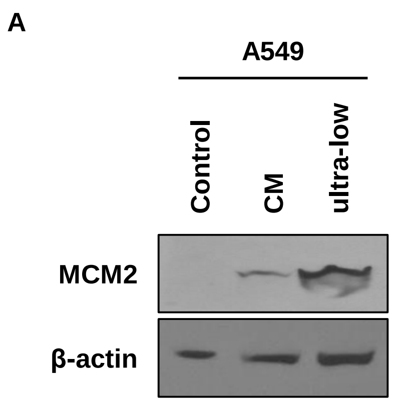

Supplymentary Figure 2. Expression of MCM2 in ultra-low attachment plates. (A) Comparison of MCM2 expression in normal culture plates and ultra-low attachment plates. Sample derive from the same experiment and that blots were processed in parallel.

Figure S3.

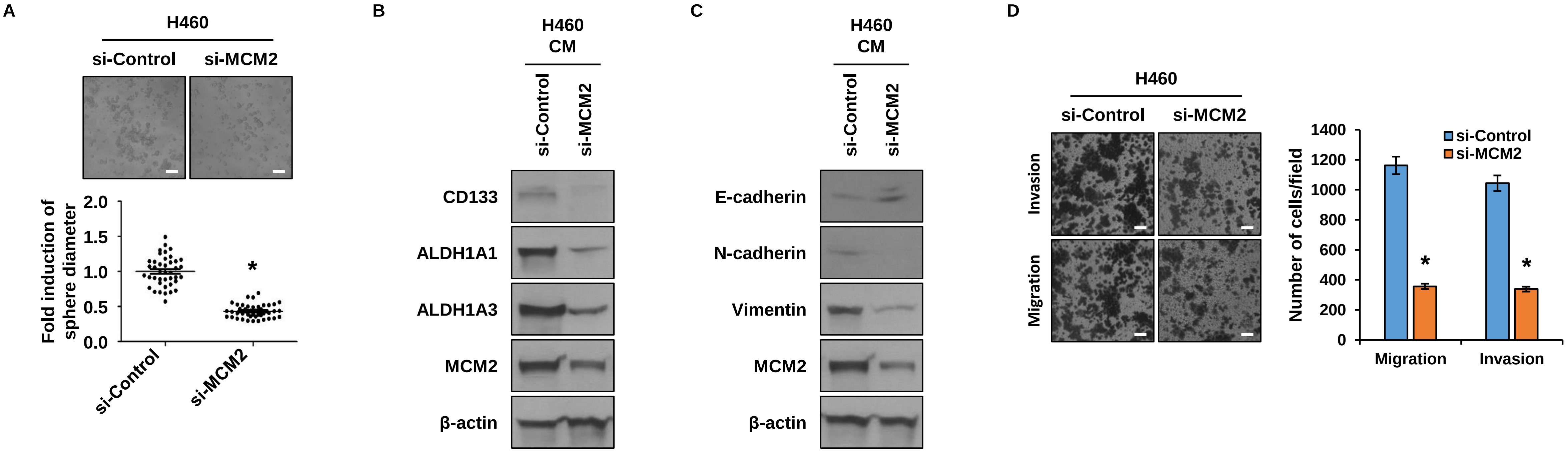

Supplymentary Figure 3. CSC and EMT effects by MCM2 in H460 lung cancer cell line. (A) Sphere-forming ability regulated by MCM2. (B) Expression of cancer stem cell marker proteins in si-RNA treated H460. (C) Expression of EMT marker proteins regulated by MCM2. (D) MCM2 regulates cell migration and invasion ability. Sample derive from the same experiment and that blots were processed in parallel. Data are presented as the mean ± standard deviation of three replicates. Scale bar, 50µm. *p<0.0005.

Figure S4.

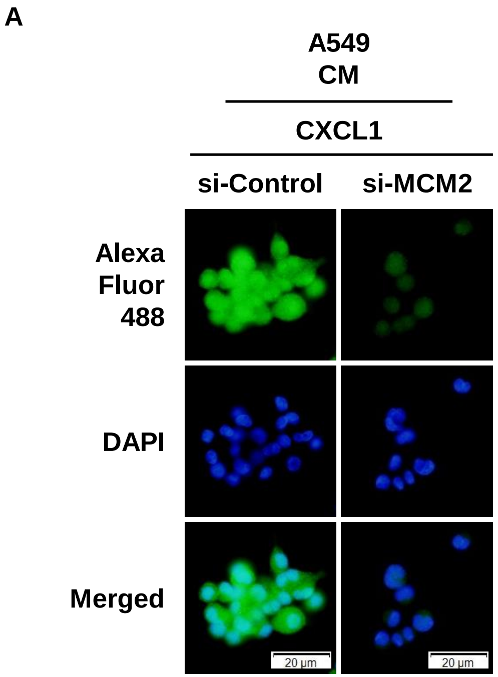

Supplymentary Figure 4. CXCL1 expression confirmed by ICC. (A) CXCL1, which is regulated depending on the expression of MCM2 in cells, was identified using ICC.

Figure S5.

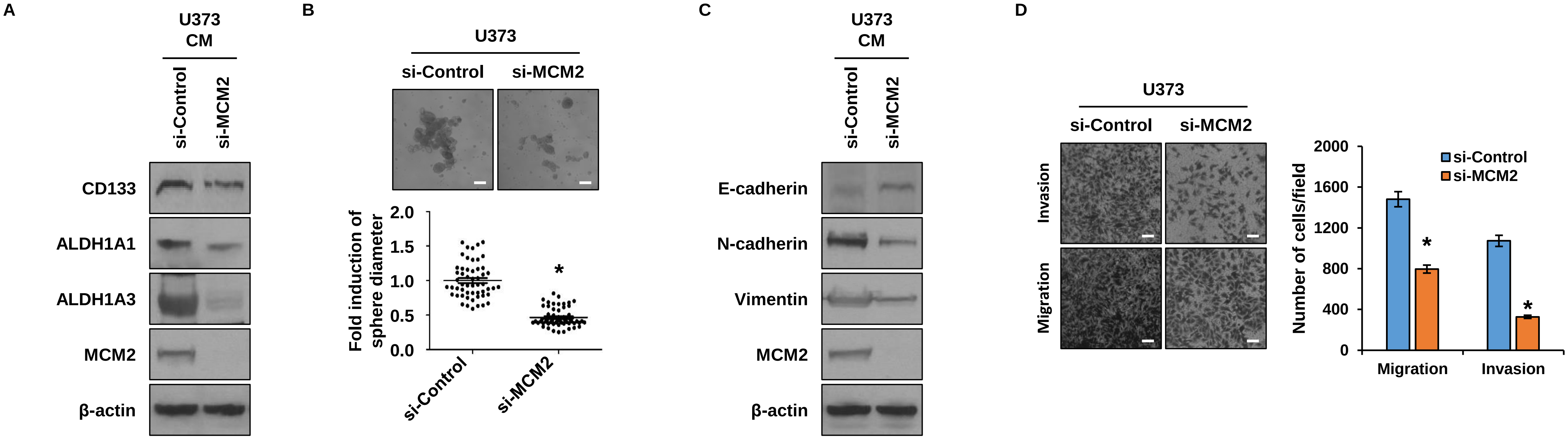

Supplymentary Figure 5. CSC and EMT regulated by MCM2 in U373 GBM cell line. (A) Cancer stem cell marker proteins decreased when MCM2 expression is suppressed. (B) Differences in sphere formation ability according to expression of MCM2 in U373 cells. (C) Cell migration and invasion ability according to differences in MCM2 expression. Sample derive from the same experiment and that blots were processed in parallel. Data are presented as the mean ± standard deviation of three replicates. Scale bar, 50μm. *$p<0.0005$.

Figure S6.

A. Uncropped blots of Figure 1A

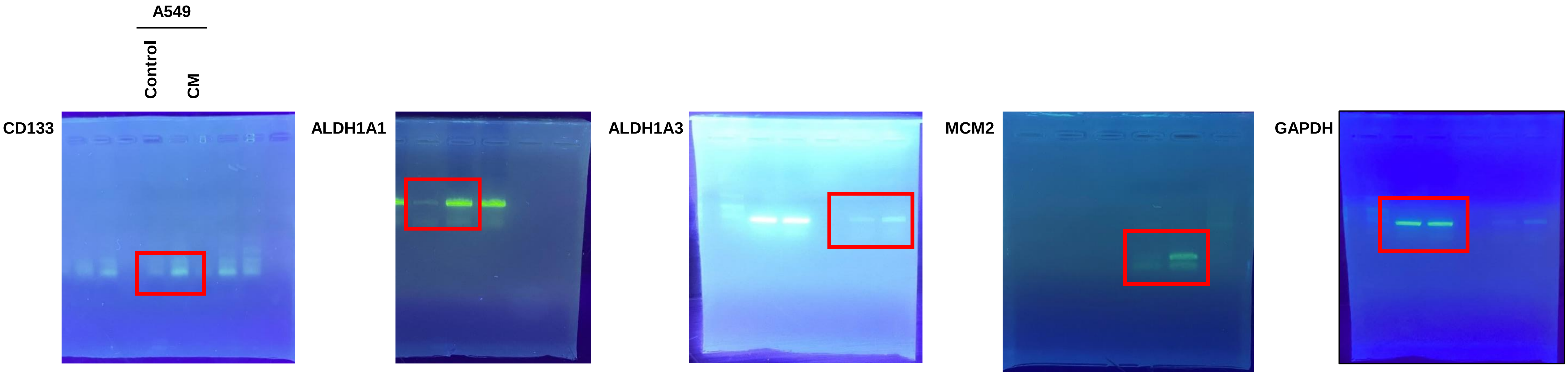

Figure S6.

B. Uncropped blots of Figure 4B

A549 CM

si-Control si-MCM2

CCL5

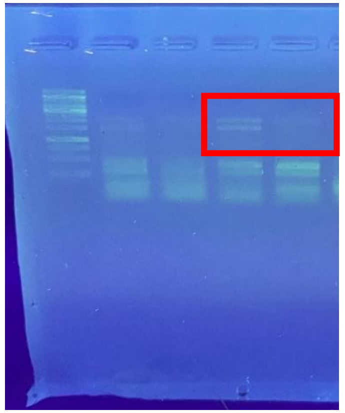

CXCL1

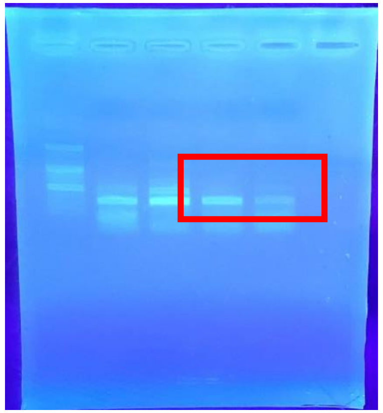

IL-18

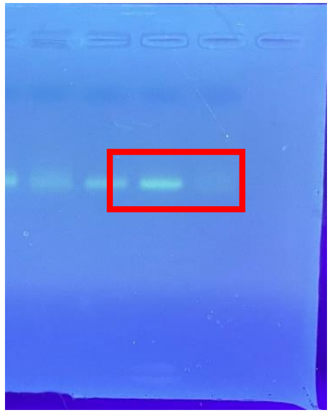

IL-21

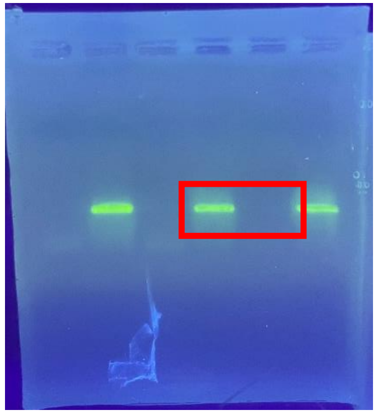

MCM2

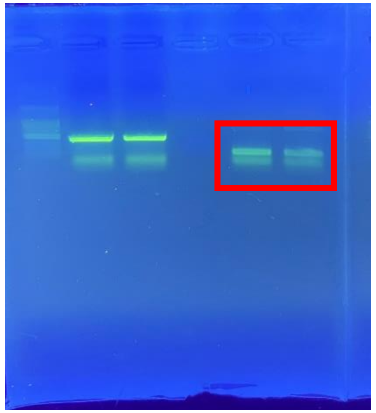

GAPDH

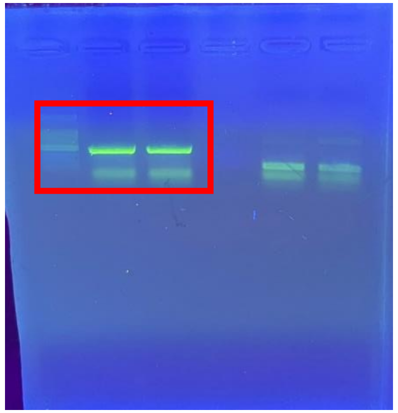

Figure S6.

C. Uncropped blots of Figure 4H

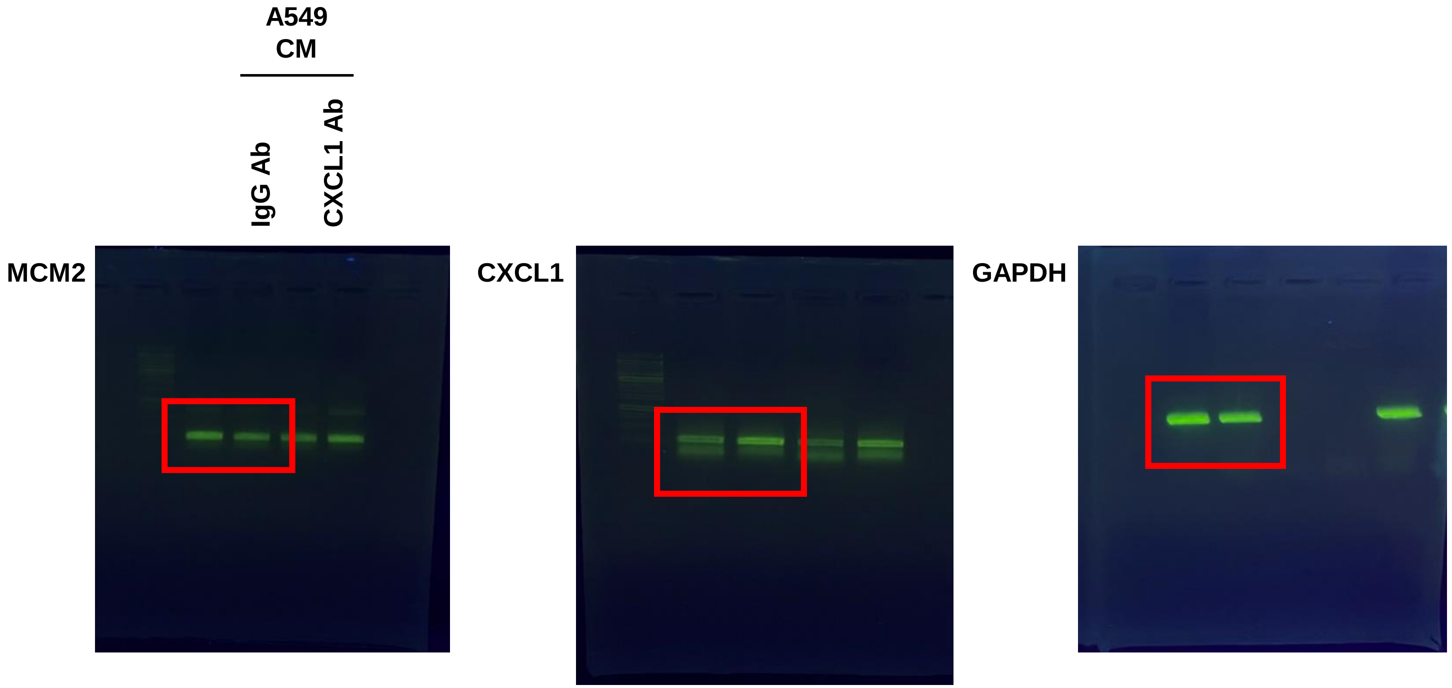

Figure S6.

D. Uncropped blots of Figure 4H

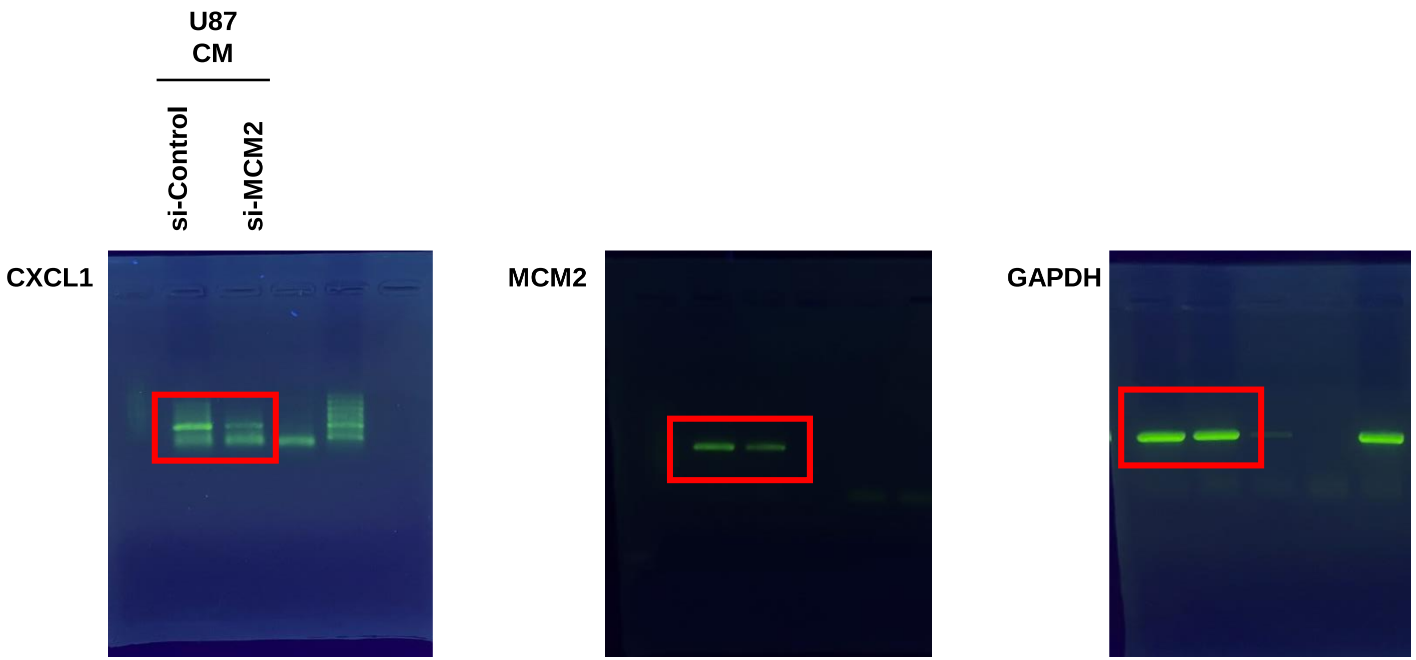

Supplymentary Figure 6. mRNA experiment raw data. (A) Uncropped blots of Figure 1A. (B) Uncropped blots of Figure 4B. (C) Uncropped blots of Figure 4H. (D) Uncropped blots of Figure 4H.

Figure S7.

A. Uncropped blots of Figure 1B

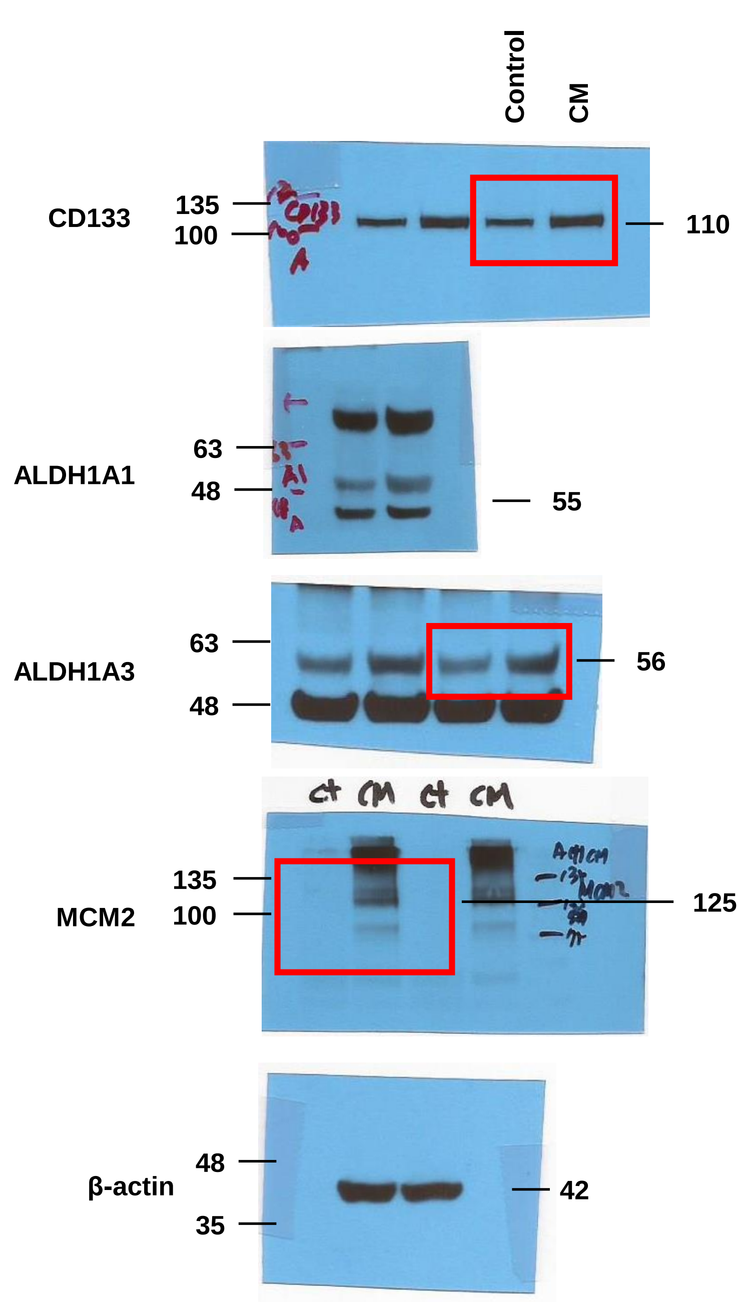

B. Uncropped blots of Figure 2D

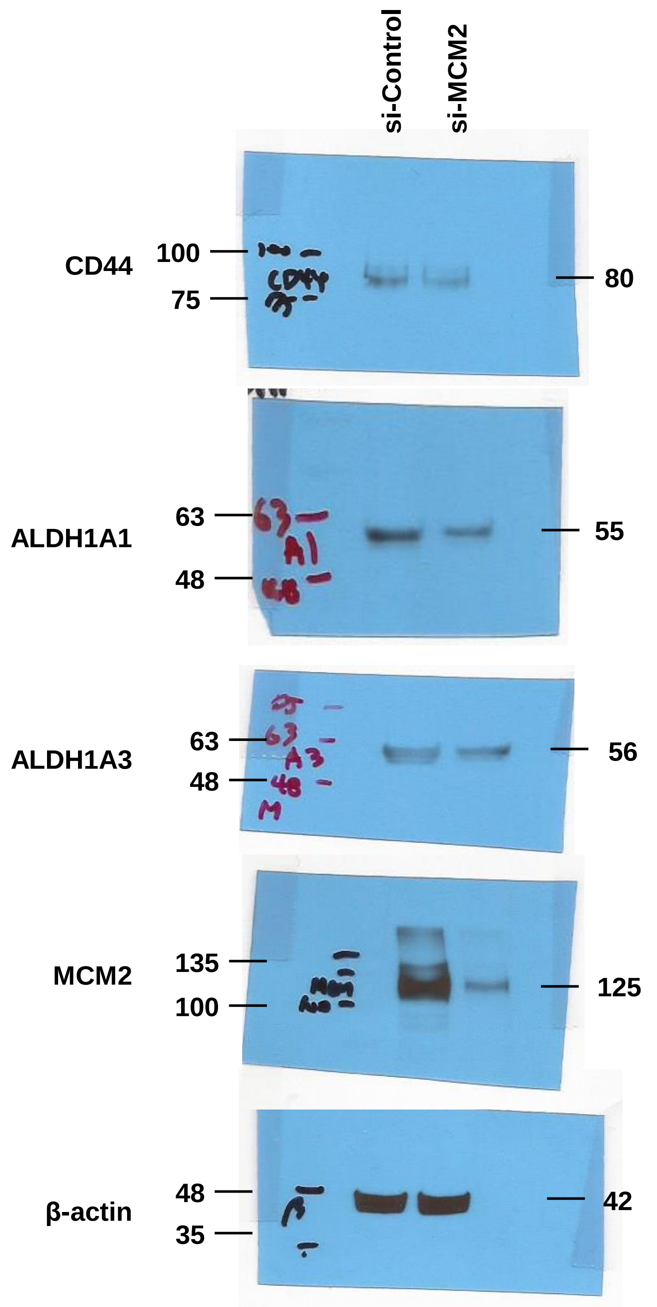

C. Uncropped blots of Figure 2E

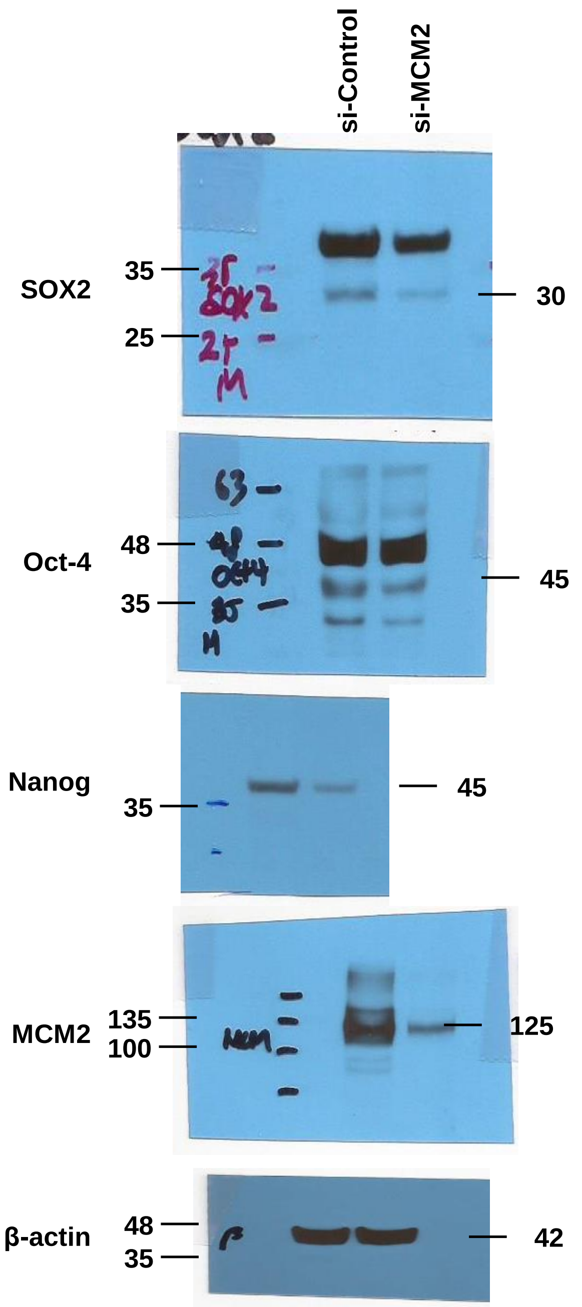

Figure S7.

D. Uncropped blots of Figure 3A

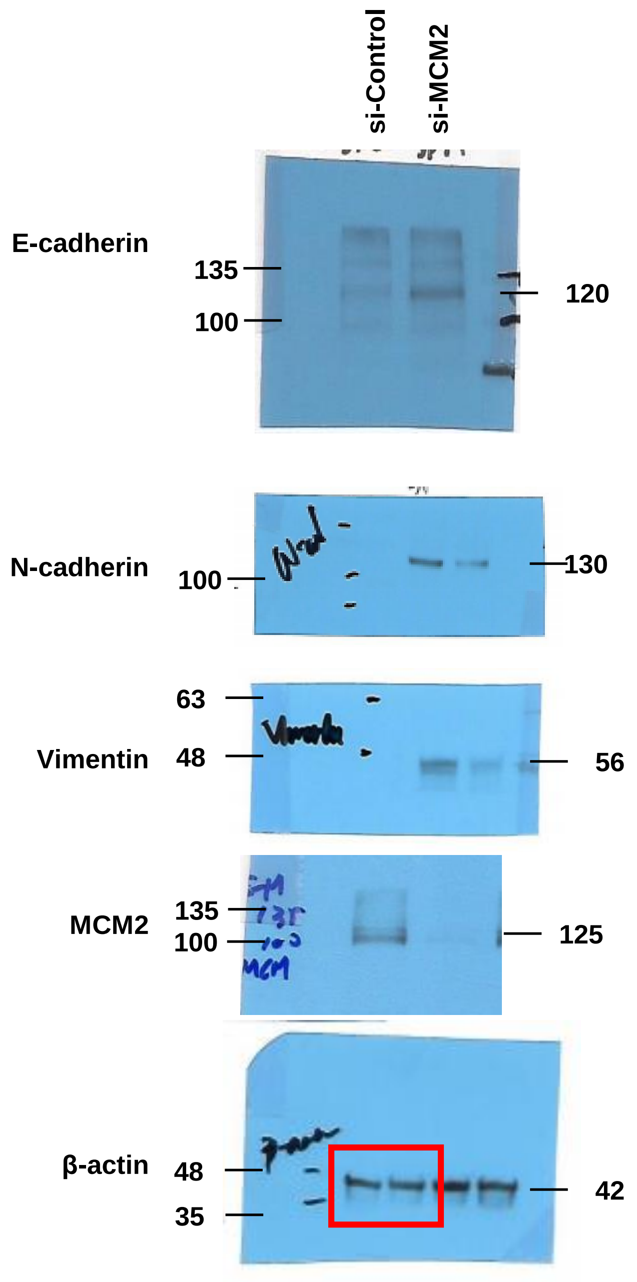

E. Uncropped blots of Figure 3B

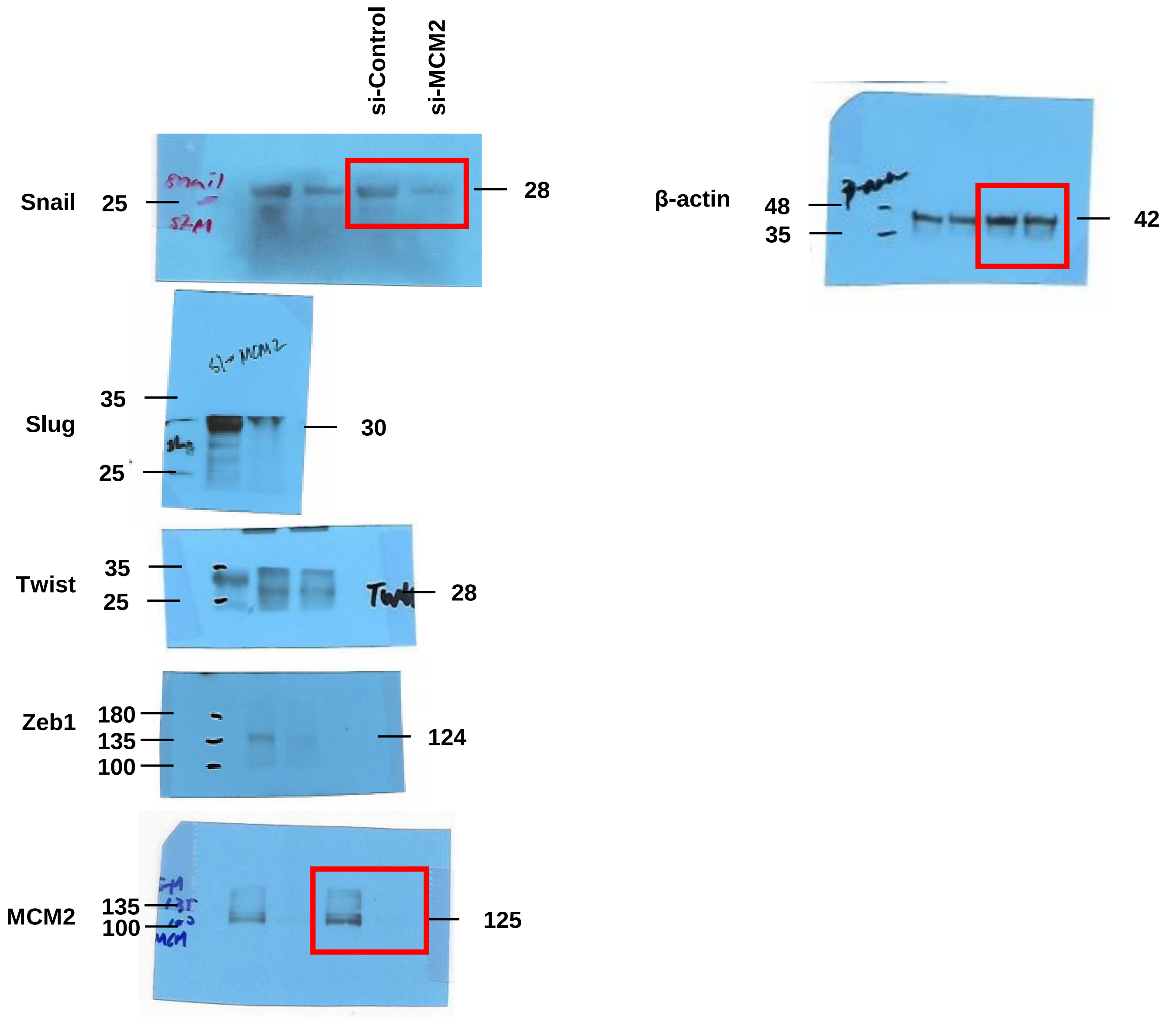

Figure S7.

F. Uncropped blots of Figure 4E

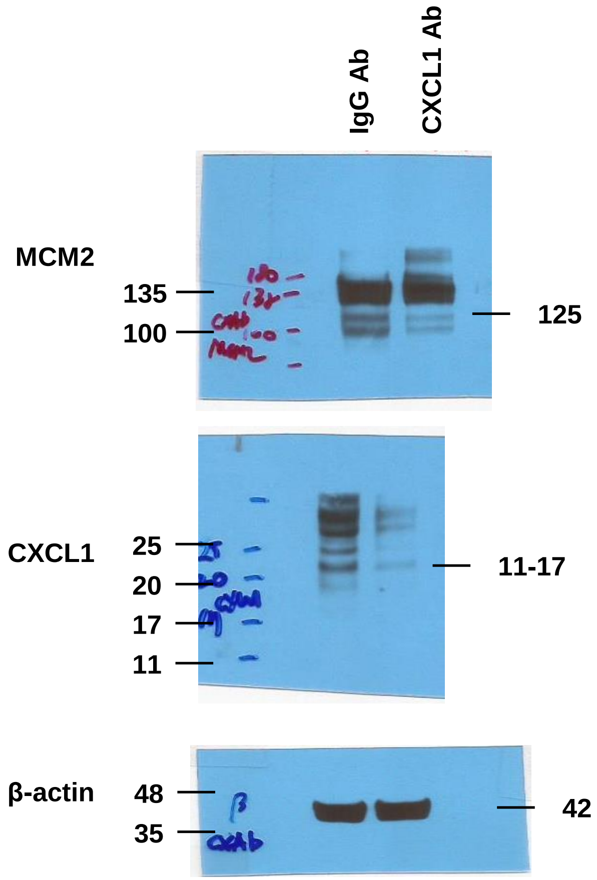

G. Uncropped blots of Figure 4G

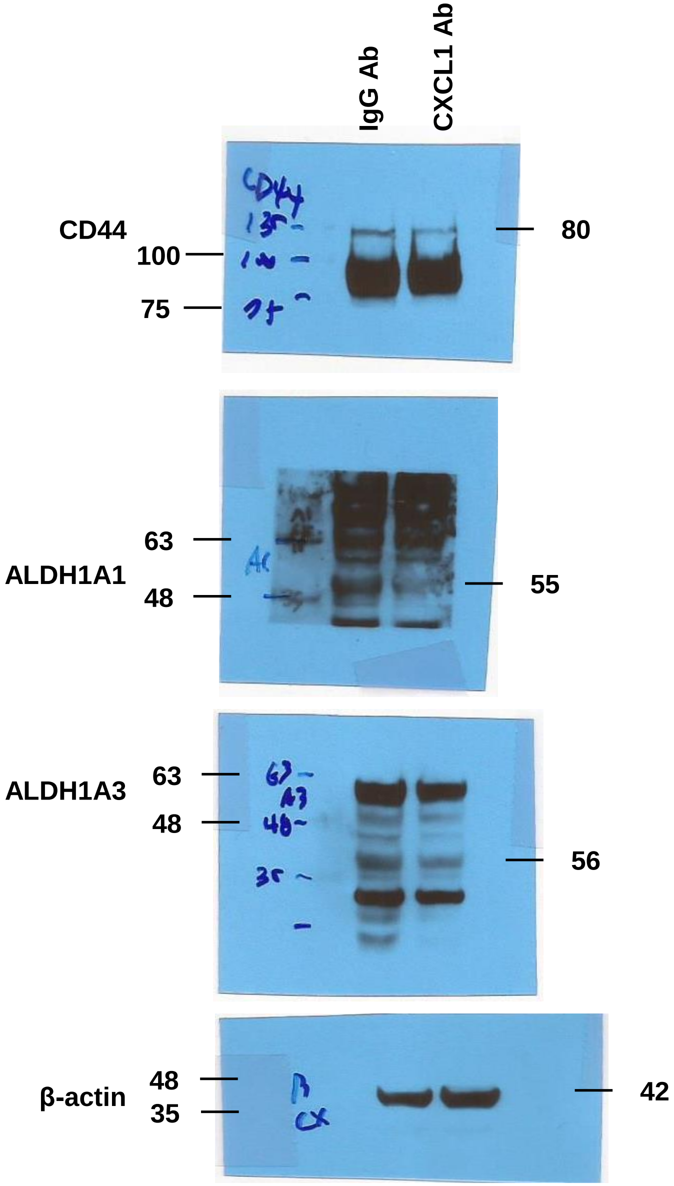

H. Uncropped blots of Figure 4H

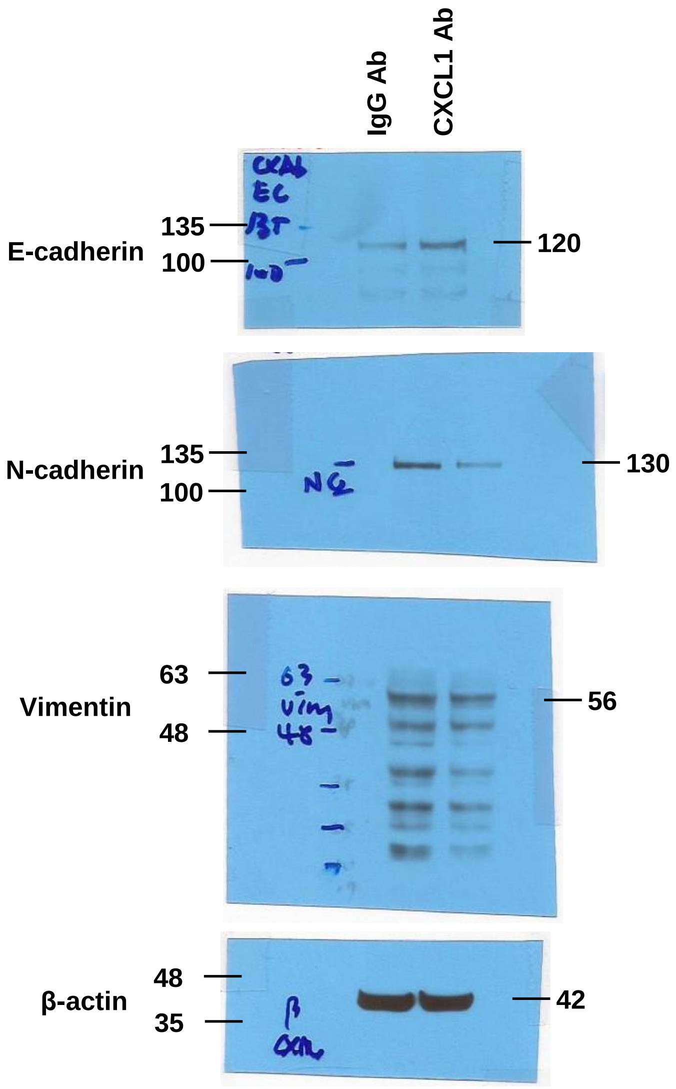

Figure S7.

I. Uncropped blots of Figure 4I

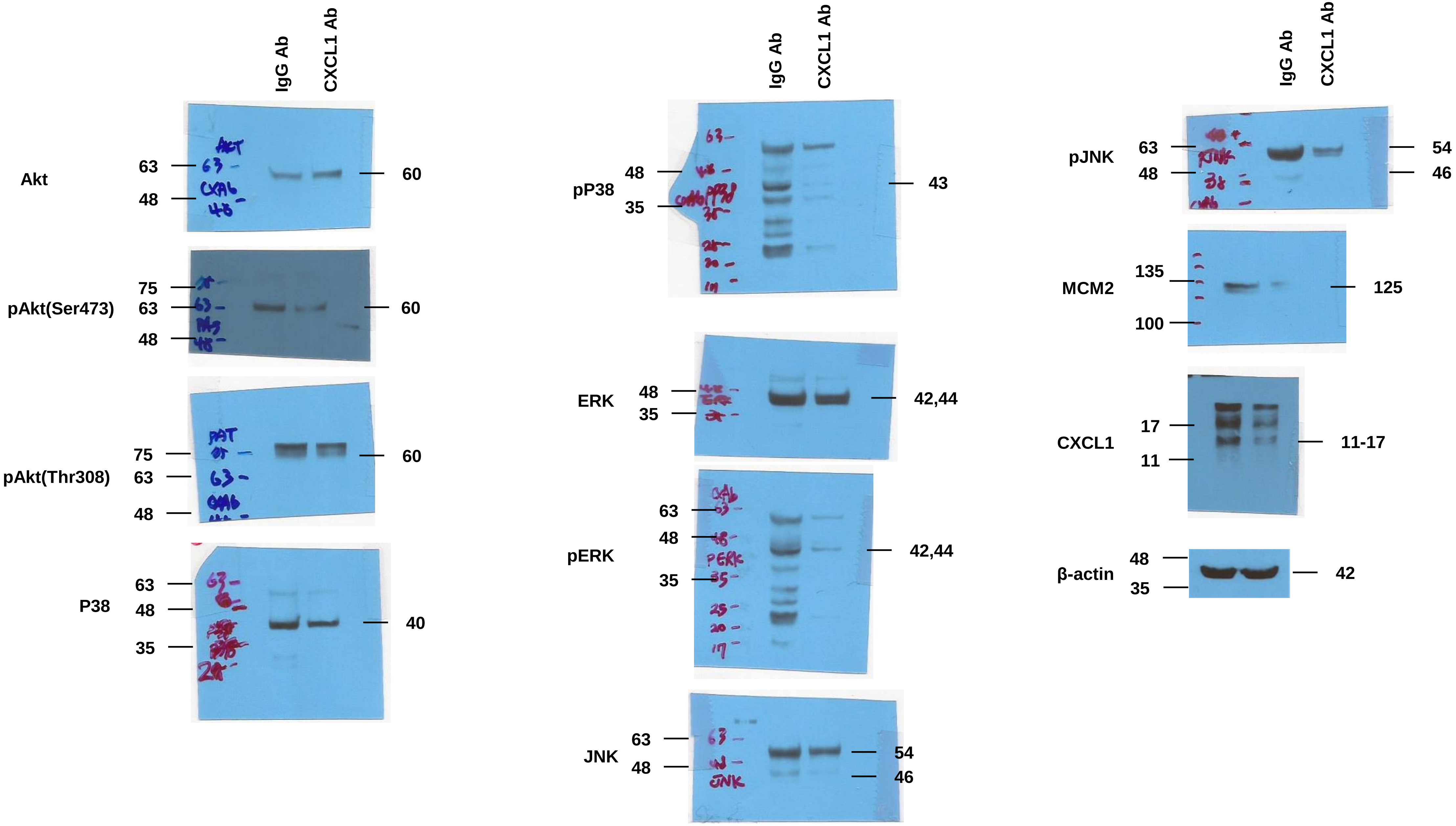

Figure S7.

J. Uncropped blots of Figure 5A

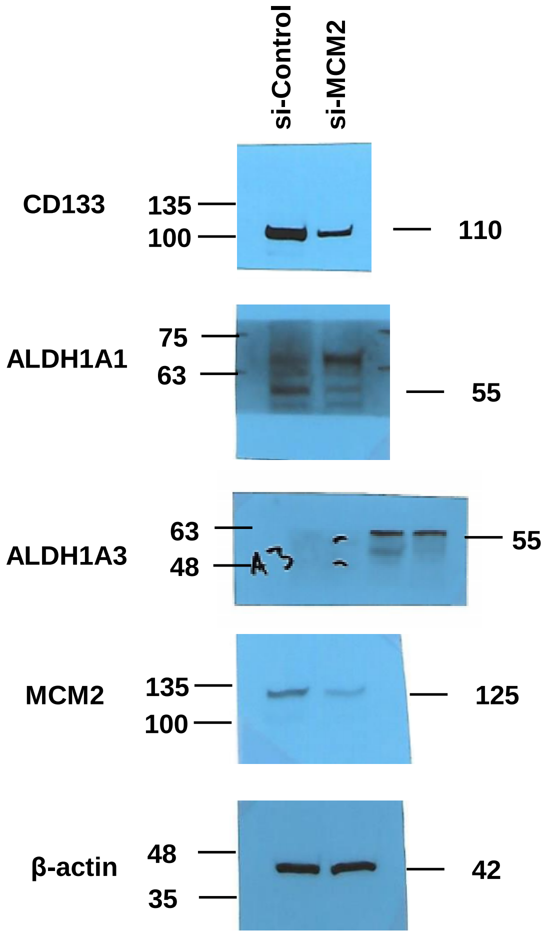

K. Uncropped blots of Figure 5D

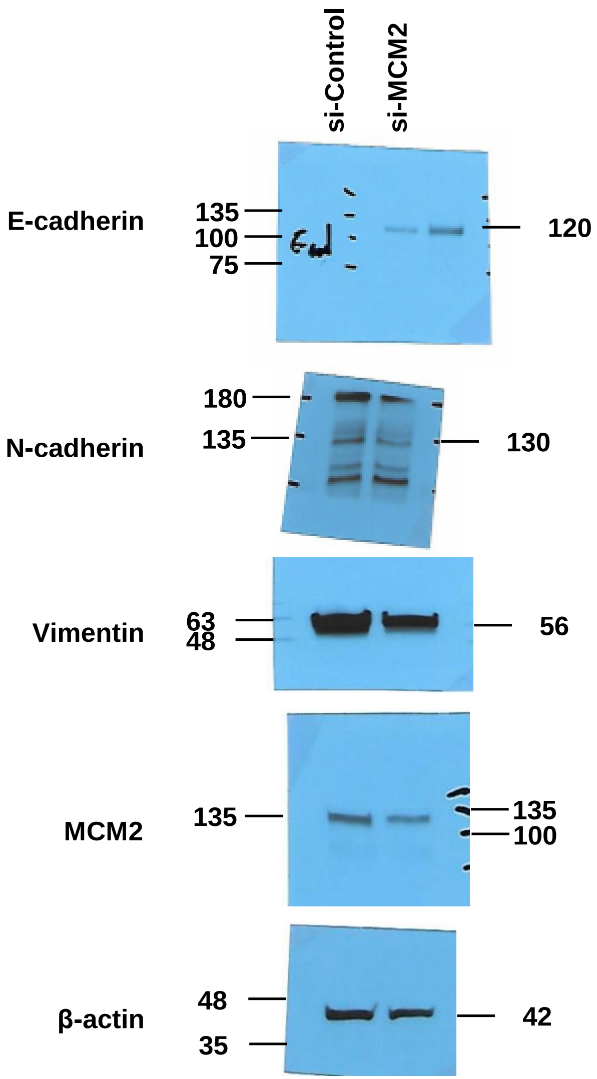

Figure S7.

L. Uncropped blots of Figure S2A

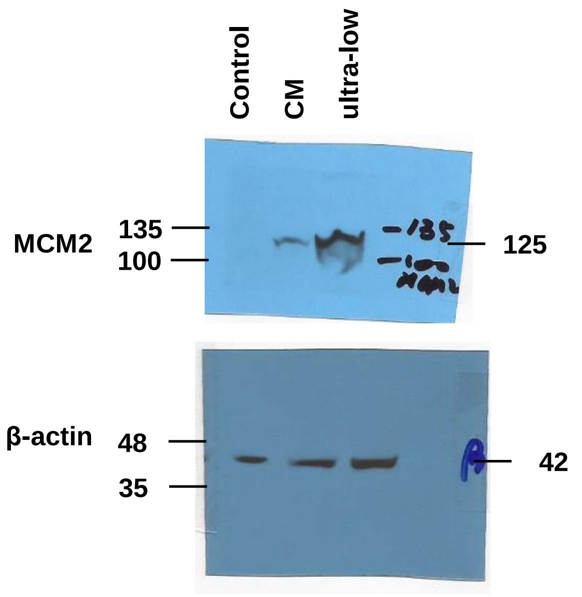

M. Uncropped blots of Figure S3A

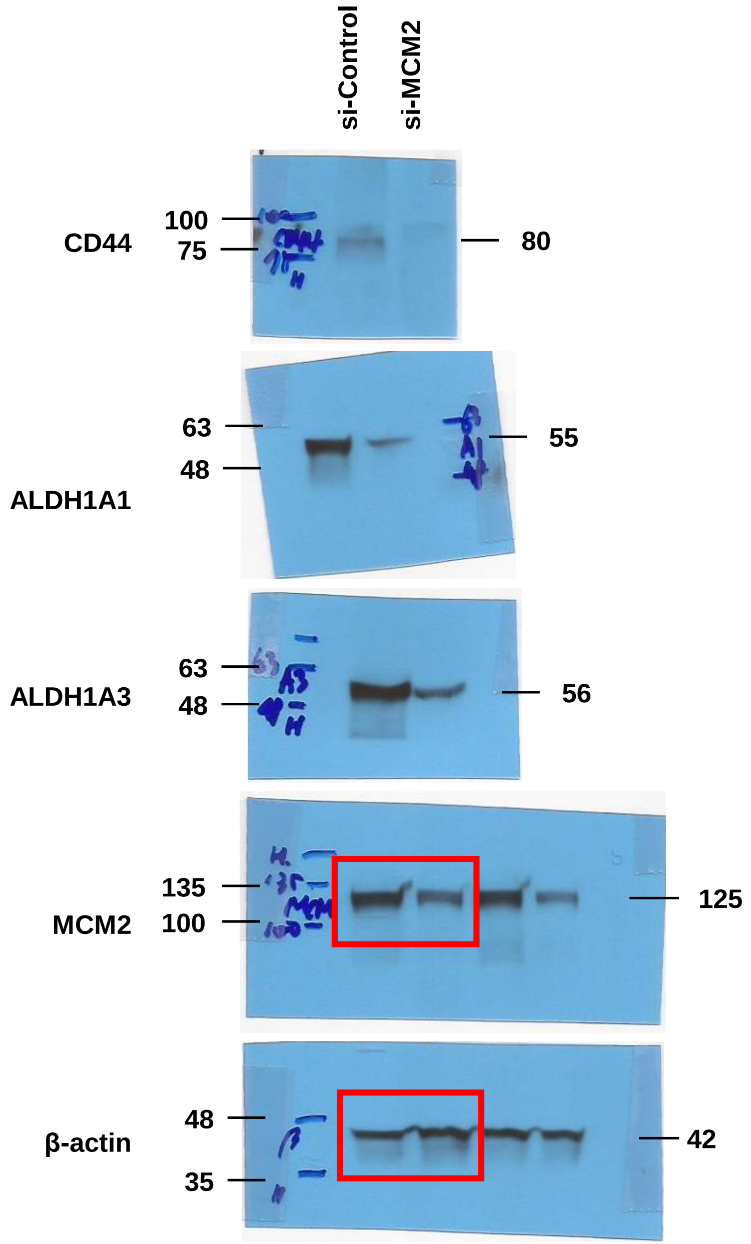

N. Uncropped blots of Figure S3C

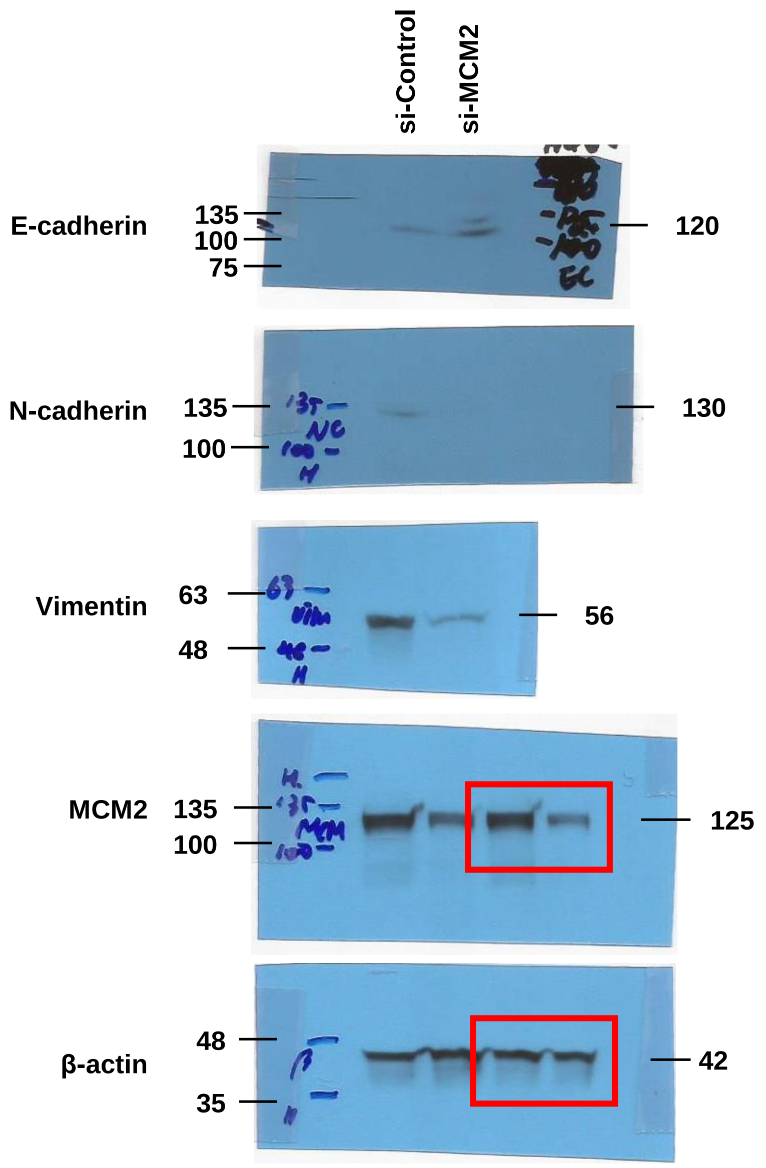

Figure S7.

O. Uncropped blots of Figure S5A

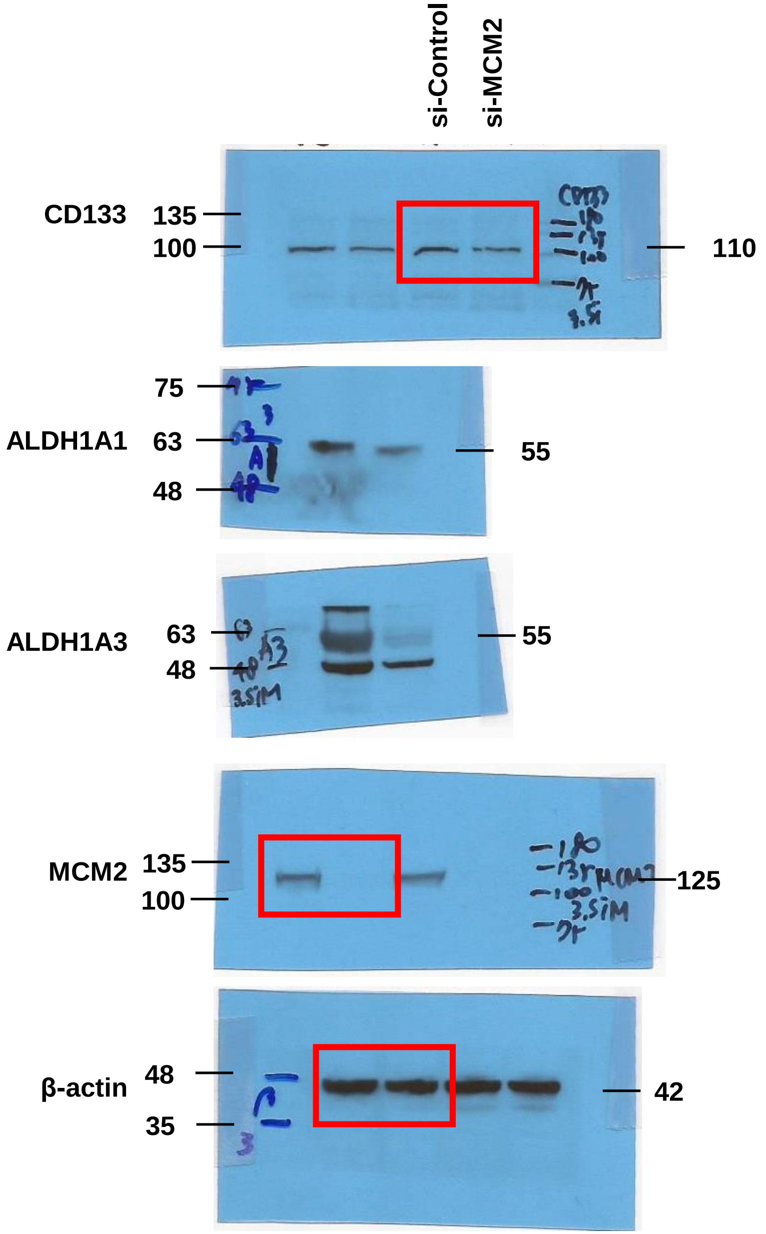

P. Uncropped blots of Figure S5C

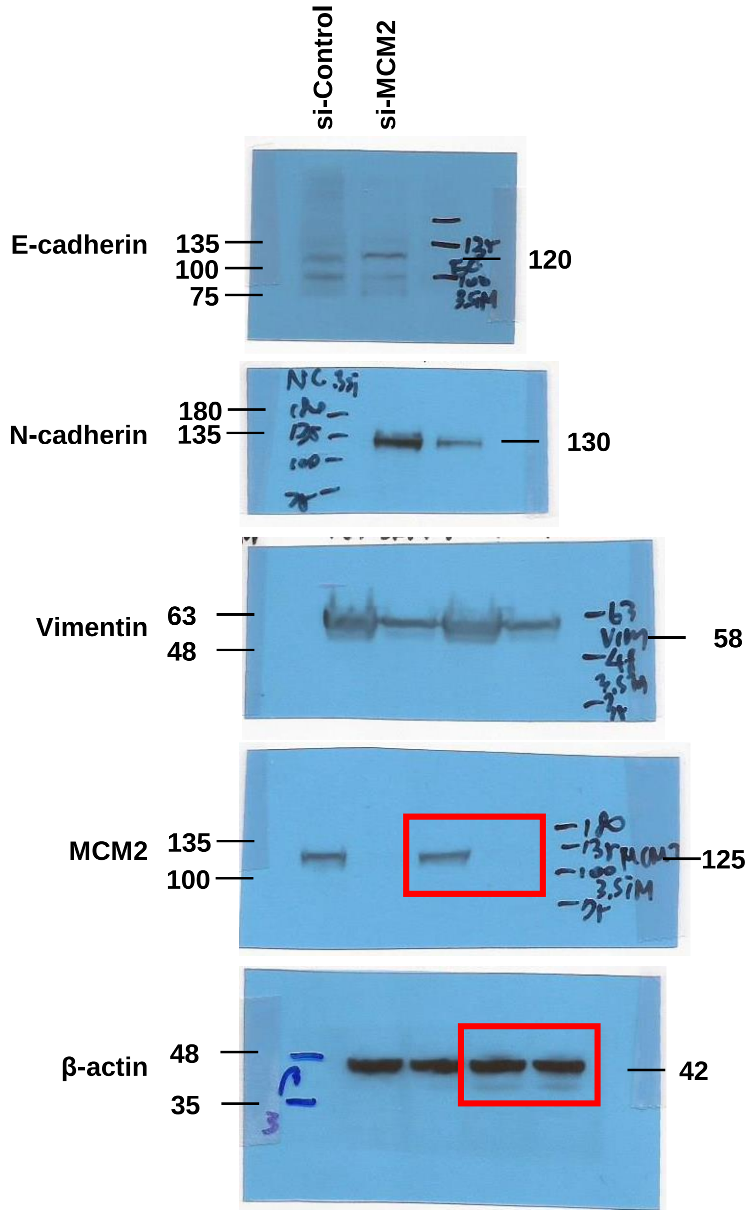

Supplymentary Figure 7. Western blots raw data. (A) Uncropped blots of Figure 1B. (B) Uncropped blots of Figure 2D. (C) Uncropped blots of Figure 2E. (D) Uncropped blots of Figure 3A. (E) Uncropped blots of Figure 3B. (F) Uncropped blots of Figure 1E. (G) Uncropped blots of Figure 4G. (H) Uncropped blots of Figure 4H. (I) Uncropped blots of Figure 4I. (J) Uncropped blots of Figure 5A. (K) Uncropped blots of Figure 5D. (L) Uncropped blots of Figure S2A. (M) Uncropped blots of Figure S3A. (N) Uncropped blots of Figure S3C. (O) Uncropped blots of Figure S5A. (P) Uncropped blots of Figure S5C.
